# Hypervalent Iodine(III)-Induced Domino Oxidative Cyclization for the Synthesis of Cyclopenta[*b*]furans

**DOI:** 10.3390/molecules21121713

**Published:** 2016-12-21

**Authors:** Mei-Huey Lin, Yu-Chun Chen, Shih-Hao Chiu, Yun-Fan Chen, Tsung-Hsun Chuang

**Affiliations:** Department of Chemistry, National Changhua University of Education, Changhua 50007, Taiwan; stu01250008@gmail.com (Y.-C.C.); shihhao9239@yahoo.com.tw (S.-H.C.); evons44@gmail.com (Y.-F.C.); th_chuang@hotmail.com (T.-H.C.)

**Keywords:** cyclopenta[*b*]furan, hypervalent iodine(III), domino oxidative cyclization, diacetoxyiodobenzene, cycloisomerization

## Abstract

A new strategy for cyclopenta[*b*]furan synthesis mediated by hypervalent iodine(III) has been described. The approach employs diacetoxyiodobenzene-induced initial dehydrogenation to a putative trienone intermediate and triggered sequential cycloisomerization to form the cyclo-penta[*b*]furan targets.

## 1. Introduction

**Scheme 1:**
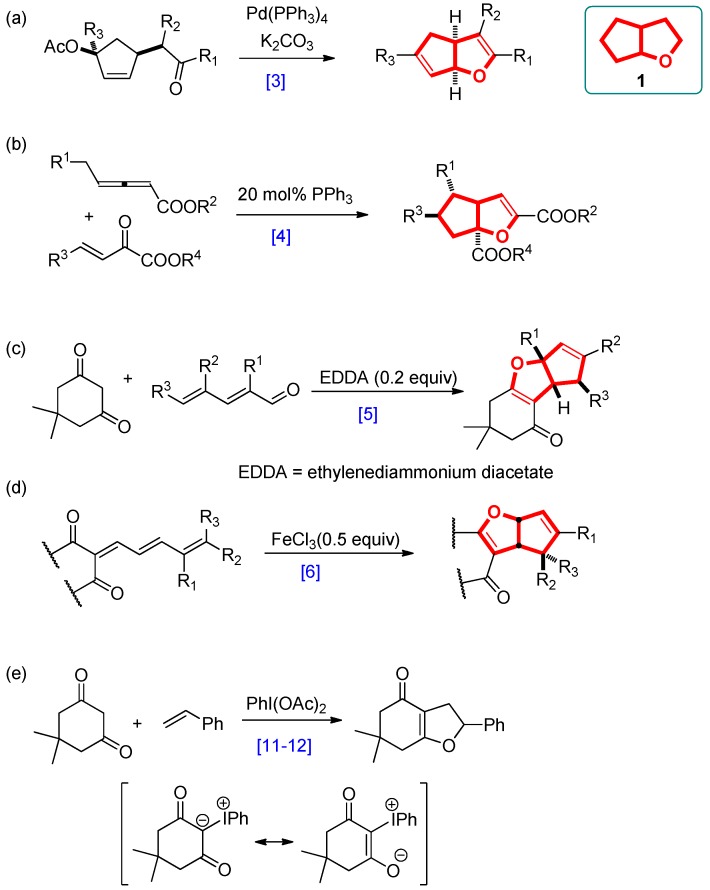
Routes for the synthesis of cyclopenta[*b*]furans.

**Scheme 2:**
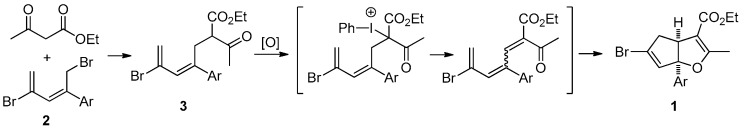
Synthetic plans for the synthesis of cyclopenta[*b*]furan derivatives **1**.

**Scheme 3:**
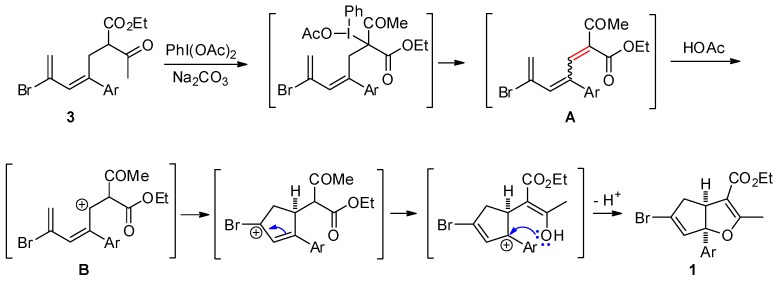
Plausible Mechanism.

Recent medicinal chemical studies have led to the identification of the cyclopenta[*b*]furan ring system as a potent inhibitor of CCR2, a chemokine receptor that plays an important role in inflammation-associated diseases [1,2]. Syntheses of the cyclopenta[*b*]furan core component have been achieved by employing palladium(0)-catalyzed intramolecular allylations (path a, Scheme 1), [3] and phosphine-catalyzed [3 + 2] cycloadditions of allenoates with β,γ-unsaturated α-ketoesters (path b, Scheme 1) [4]. In addition, the construction of tricyclic cyclopenta[*b*]furan derivatives was elaborated through ethylenediammonium diacetate (EDDA)-catalyzed tandem Knoevenagel-polycyclization reactions between 1,3-dicarbonyl compounds and α,β,γ,δ-unsaturated aldehydes (path c, Scheme 1) or Lewis acid-promoted cycloisomerization of conjugated trienones (path d, Scheme 1) [5,6]. On the other hand, hypervalent hypervalent iodine species possess similar chemical properties and reactivity as heavy metal reagents but lower toxicity and an environmentally benign nature. Therefore, the utilization of hypervalent iodine in organic synthesis has been drawing intensive attention [7,8,9,10,11,12]. Among its reactions, the reactions of 1,3-dicarbonyl compounds with hypervalent iodine(III) species such as diacetoxyiodobenzene generate β-diketo iodonium ylides which behave as a 1,3-dipole, with an electrophilic center next to a carbonyl group. For example, the synthetic application of hypervalent iodine(III) in forming heterocycles involves the addition of β-diketo iodonium ylides to olefins to form fused dihydrofurans (path e, Scheme 1) [13,14]. Recent progress in the use of hypervalent iodine(III) reagents in organic synthesis has shown that diacetoxyiodobenzene-mediated dehydrogenation reactions of α-alkyl-β-dicarbonyl compounds provide an alternative route to the Knoevenagel condensation [15,16]. Herein, we have devised a route for employing hypervalent iodine(III) in the synthesis of cyclopenta[*b*]furan derivatives **1** (Scheme 2).

## 2. Results and Discussions

In the study described below, we have explored a method for the synthesis of cyclopenta[*b*]furans containing a stereogenic, quaternary, aryl-substituted bridgehead carbon. For the preparation of 2-aryl-1,4-dibromopenta-2,4-dienes **2** we used a literature procedure developed in our group [17]. The β-ketoesters linked with pentadiene tether **3** were prepared by *C*-alkylation of ethyl acetoacetate enolate with **2**; however, the chromatographic purification of compounds **3** is difficult in most cases (Table 1). In fact, only **3h** can be isolated in pure form by recrystallization after chromatographic purification. Crude analogues **3a**–**g** as obtained after silica-gel plug treatment were carried directly into the next step, therefore, purity and exact yields of **3a**–**g** are not given in Table 1. In order to evaluate the feasibility of the new strategy for cyclopenta[*b*]furan synthesis, **3b** serves as effective substrate in the reaction with diacetoxyiodobenzene that takes place by a one-pot, initial dehydrogenation to give a putative trienone intermediate and sequential cycloisomerization to form the cyclopenta[*b*]furan (Table 2). The results show that the putative trienone produced in these reactions is not isolable using chromatography, and at ambient temperature only the formation of the trienone was observed (Table 2, entries 1 and 8). At elevated temperature, the putative trienone underwent cycloisomerization to form cyclopenta[*b*]furans (entry 1 vs. entry 2, entry 7 vs. entry 8), reactions in which inorganic bases gave better yields than organic bases (entry 4 vs. entry 5, entry 8 vs. entry 9). In addition, the oxidation-cycloisomerization process happened smoothly when [bis(trifluoroacetoxy)iodo]benzene was employed. The ideal conditions for this process involve the use of diacetoxyiodobenzene and sodium carbonate in refluxing ethanol (Table 2, entry 7). Next, a variety of aryl-substituted substrates **3** were examined and Suzuki reactions were pursued in order to confirm the structural determination and the relative stereochemistry at the ring junction of the cyclopenta[*b*]furans **1** (Table 1).

**Figure 1:**
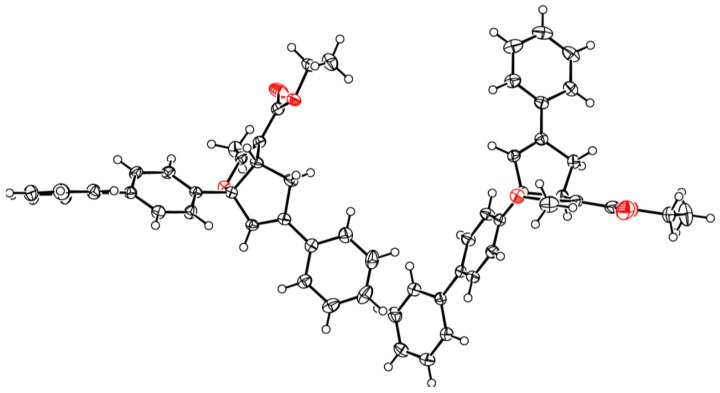
ORTEP plot of X-ray crystallographic data for **4b**.

The stereochemistry of the fused-bicyclic ring system in **4b** was shown to be *cis* using X-ray crystallographic analysis (Figure 1 and Supplementary Material) [18].

A proposed plausible mechanism is shown in Scheme 3. First, the formation of putative trienone **A** through diacetoxyiodobenzene-mediated dehydrogenation reaction gave the newly formed C–C double bond as *E*/*Z*-form mixtures or ones where the *Z*-isomer predominated [15]. However, only the *E*-isomer possesses the proper steric alignment to form the target cyclopenta[*b*]furans **1**. Therefore, a simple thermal cycloisomerization of the trienone to a cyclopenta[*b*]furan is not likely in this scenario. In the diacetoxyiodobenzene-mediated dehydrogenation process at elevated temperature, acetic acid produced during the reaction presumably plays an important role in promoting the sequential cycloisomerization from a cationic dienyl structure **B** to form the cyclopenta[*b*]furans.

## 3. Materials and Methods 

### 3.1. General Information

All commercially available chemicals were used without further purification. TLC analyses were run on a TLC glass plate (silica gel 60 F254, EMD Millipore, Darmstadt, Germany) and were visualized using UV and a solution of phosphomolybdic acid in ethanol (5 wt %) or *p*-anisaldehyde stain. Flash chromatography was performed using silica gel (70–230 mesh, EMD Millipore). ^1^H-NMR spectra were recorded on a 300 MHz spectrometer (Bruker AV-300, Bruker BioSpin GmbH, Karlsruhe, Germany). ^13^C-NMR spectra were recorded at 75 MHz on the same instrument with complete proton decoupling (see Supplementary Material). Chemical shifts are reported relative to CHCl_3_ [δ_H_ 7.24, δ_C_ (central line) 77.0]. Mass spectra and high-resolution mass spectra were recorded on the Thermo/Finnigan Quest MAT Mass Spectrometer (Thermo Finnigan LLC, San Jose, CA, USA).

### 3.2. Synthesis

#### 3.2.1. General Procedure for the Synthesis of Cyclopenta[*b*]furans **1**

A mixture of bromodiene **2** (0.3 mmol), ethyl acetoacetate (0.3 mmol, 39 mg), and NaOEt (0.4 mmol, 27 mg) in EtOH (2 mL) was stirred at reflux overnight. The mixture was cooled to ambient temperature (25–28 °C) and transferred to a separatory funnel, followed by addition of Et_2_O (5 mL) and water (10 mL). The aqueous layer was back extracted with Et_2_O (2 mL × 2). The combined organic layers were washed with brine (5 mL), dried over MgSO_4_, and concentrated in a rotary evaporator. The residue was purified by a silica gel plug using Et_2_O/hexanes (10/1, 10 mL) as the eluent, followed by concentration to give the bromoester **3**. To a solution of bromoester **3** (0.3 mmol, based on bromo-diene **2**) in EtOH (1.2 mL) was added Na_2_CO_3_ (0.6 mmol, 64 mg) and PhI(OAc)_2_ (0.6 mmol, 193 mg) at ambient temperature. The resulting mixture was heated to reflux under N_2_ for 3 h. The reaction was then cooled room temperature and H_2_O (2 mL) was added. Et_2_O (5 mL) was added and the mixture was transferred to a separatory funnel. The aqueous layer was back extracted with Et_2_O (5 mL × 2). The combined organic layers were washed with 2 N HCl (2 mL), dried over Na_2_SO_4_, filtered, and concentrated in a rotary evaporator. The residue was purified by silica gel chromatography using Et_2_O/hexanes (1/10) as eluent to give the title products as yellow oils. Using this general procedure, the following title compounds were obtained:

*Ethyl 5-bromo-2-methyl-6a-phenyl-4,6a-dihydro-3aH-cyclopenta[b]furan-3-carboxylate* (**1a**). Yield 55 mg (72%). TLC (Et_2_O/hexanes (1:10)) *R*_f_ = 0.44; ^1^H-NMR (CDCl_3_) δ 1.25 (t, *J* = 7.2 Hz, 3H), 2.28 (d, *J* = 1.5 Hz, 3H), 2.83 (dt, *J* = 17.4, 2.1 Hz, 1H), 3.15 (ddd, *J* = 17.4, 7.8, 2.1 Hz, 1H), 3.74 (dt, *J* = 7.8, 1.5 Hz, 1H), 4.09–4.20 (m, 2H), 5.98 (t, *J* = 2.1 Hz, 1H), 7.25–7.35 (m, 5H); ^13^C-NMR (CDCl_3_) δ 14.3 (CH_3_), 14.4 (CH_3_), 46.9 (CH_2_), 53.9 (CH), 59.6 (CH_2_), 100.8 (C) 105.8 (C), 124.5 (CH × 2), 128.0 (CH), 128.6 (CH × 2), 129.1 (C), 131.9 (CH), 142.0 (C), 165.5 (C), 167.0 (C); IR (neat) 2977, 1698, 1644 cm^−1^; EI-MS *m*/*z* (rel intensity) 350 ([M + 2]^+^, 10), 348 ([M]^+^, 10), 302 (29), 223 (100); HRMS [M]^+^ calcd. for C_17_H_17_BrO_3_: 348.0361, found 348.0366.

*Ethyl 5-bromo-6a-(4-bromophenyl)-2-methyl-4,6a-dihydro-3aH-cyclopenta[b]furan-3-carboxylate* (**1b**). Yield 100 mg (78%). TLC (Et_2_O/hexanes (1:10)) *R*_f_ = 0.43; ^1^H-NMR (CDCl_3_) δ 1.26 (t, *J* = 7.2 Hz, 3H), 2.28 (d, *J* = 1.5 Hz, 3H), 2.80 (dt, *J* = 17.4, 1.8 Hz, 1H), 3.14 (ddd, *J* = 17.4, 7.8, 1.8 Hz, 1H), 3.69 (dt, *J* = 7.8, 1.5 Hz, 1H), 4.06–4.28 (m, 2H), 5.93 (t, *J* = 1.5 Hz, 1H), 7.15 (d, *J* = 7.8 Hz, 2H), 7.46 (d, *J* = 7.8 Hz, 2H); ^13^C-NMR (CDCl_3_) δ 14.2 (CH_3_), 14.4 (CH_3_), 46.8 (CH_2_), 53.9 (CH), 59.7 (CH_2_), 100.3 (C) 105.9 (C), 122.0 (C), 126.3 (CH × 2), 129.6 (C), 131.5 (CH), 131.7 (CH × 2), 141.1 (C), 165.4 (C), 166.8 (C); IR (neat) 2976, 1703, 1488 cm^−1^; EI-MS *m*/*z* (rel intensity) 428 ([M + 2]^+^, 19), 426 ([M]^+^, 10), 382 (59), 301 (100); HRMS [M]^+^ calcd. for C_17_H_16_Br_2_O_3_: 425.9466, found 425.9462.

*Ethyl 5-bromo-6a-(4-chlorophenyl)-2-methyl-4,6a-dihydro-3aH-cyclopenta[b]furan-3-carboxylate* (**1c**). Yield 104 mg (90%). TLC (Et_2_O/hexanes (1:10)) *R*_f_ = 0.43; ^1^H-NMR (CDCl_3_) δ 1.25 (t, *J* = 7.2 Hz, 3H), 2.28 (d, *J* = 1.5 Hz, 3H), 2.83 (dt, *J* = 17.4, 2.1 Hz, 1H), 3.13 (ddd, *J* = 17.4, 7.8, 2.1 Hz, 1H), 3.68 (dt, *J* = 7.8, 1.5 Hz, 1H), 4.09–4.20 (m, 2H), 5.93 (t, *J* = 2.1 Hz, 1H), 7.20 (d, *J* = 8.7 Hz, 2H), 7.31 (d, *J* = 8.7 Hz, 2H); ^13^C-NMR (CDCl_3_) δ 14.2 (CH_3_), 14.3 (CH_3_), 46.8 (CH_2_), 53.9 (CH), 59.6 (CH_2_), 100.2 (C) 105.9 (C), 125.9 (CH × 2), 128.7 (CH × 2), 129.5 (C), 131.5 (CH), 133.8 (C), 140.5 (C), 165.3 (C), 166.7 (C); IR (neat) 2977, 1700, 1646 cm^−1^; EI-MS *m*/*z* (rel intensity) 384 ([M + 2]^+^, 15), 382 ([M]^+^, 12), 338 (44), 257 (100); HRMS [M]^+^ calcd. for C_17_H_16_BrClO_3_: 381.9971, found 381.9978.

*Ethyl 5-bromo-2-methyl-6a-(p-tolyl)-4,6a-dihydro-3aH-cyclopenta[b]furan-3-carboxylate* (**1d**). Yield 76 mg (70%). TLC (Et_2_O/hexanes (1:10)) *R*_f_ = 0.51; ^1^H-NMR (CDCl_3_) δ 1.25 (t, *J* = 7.2 Hz, 3H), 2.28 (d, *J* = 1.5 Hz, 3H), 2.32 (s, 3H), 2.82 (dt, *J* = 17.4, 1.8 Hz, 1H), 3.13 (ddd, *J* = 17.4, 7.8, 1.8 Hz, 1H), 3.72 (dt, *J* = 7.8, 1.5 Hz, 1H), 4.09–4.20 (m, 2H), 5.97 (t, *J* = 1.8 Hz, 1H), 7.15 (s, 4H); ^13^C-NMR (CDCl_3_) δ 14.3 (CH_3_), 14.4 (CH_3_), 21.0 (CH_3_), 46.8 (CH_2_), 53.8 (CH), 59.5 (CH_2_), 100.7 (C) 105.8 (C), 124.4 (CH × 2), 128.9 (C), 129.2 (CH × 2), 131.9 (CH), 137.8 (C), 139.0 (C), 165.6 (C), 167.0 (C); IR (neat) 2979, 1702, 1644 cm^−1^; EI-MS *m*/*z* (rel intensity) 364 ([M + 2]^+^, 5), 362 ([M]^+^, 6), 316 (19), 237 (100); HRMS [M]^+^ calcd. for C_18_H_19_BrO_3_: 362.0518, found 362.0515.

*Ethyl 5-bromo-6a-(4-methoxyphenyl)-2-methyl-4,6a-dihydro-3aH-cyclopenta[b]furan-3-carboxylate* (**1e**). Yield 74 mg (65%). TLC (Et_2_O/hexanes (1:10)) *R*_f_ = 0.48; ^1^H-NMR (CDCl_3_) δ 1.25 (t, *J* = 7.2 Hz, 3H), 2.27 (d, *J* = 1.5 Hz, 3H), 2.81 (dt, *J* = 17.4, 1.8 Hz, 1H), 3.12 (ddd, *J* = 17.4, 7.8, 1.8 Hz, 1H), 3.71 (dt, *J* = 7.8, 1.5 Hz, 1H), 3.78 (s, 3H), 4.06–4.22 (m, 2H), 5.97 (t, *J* = 1.8 Hz, 1H), 6.85 (d, *J* = 8.7 Hz, 2H), 7.18 (d, *J* = 8.7 Hz, 2H); ^13^C-NMR (CDCl_3_) δ 14.3 (CH_3_), 14.4 (CH_3_), 46.8 (CH_2_), 53.7 (CH), 55.3 (CH_3_), 59.5 (CH_2_), 100.6 (C), 105.8 (C), 113.9 (CH × 2), 125.8 (CH × 2), 128.8 (C), 131.9 (CH), 134.1 (C), 159.3 (C), 165.6 (C), 166.9 (C); IR (neat) 2981, 1698, 1643 cm^−1^; EI-MS *m*/*z* (rel intensity) 380 ([M + 2]^+^, 6), 378 ([M]^+^, 6), 332 (20), 253 (100); HRMS [M]^+^ calcd. for C_18_H_19_BrO_4_: 378.0467, found 378.0458.

*Ethyl 5-bromo-6a-(3-methoxyphenyl)-2-methyl-4,6a-dihydro-3aH-cyclopenta[b]furan-3-carboxylate* (**1f**). Yield 72 mg (63%). TLC (Et_2_O/hexanes (1:10)) *R*_f_ = 0.45; ^1^H-NMR (CDCl_3_) δ 1.24 (t, *J* = 7.2 Hz, 3H), 2.28 (d, *J* = 1.5 Hz, 3H), 2.82 (dt, *J* = 17.4, 1.8 Hz, 1H), 3.14 (ddd, *J* = 17.4, 7.8, 1.8 Hz, 1H), 3.73 (dt, *J* = 7.8, 1.8 Hz, 1H), 3.79 (s, 3H), 4.03–4.24 (m, 2H), 5.95 (t, *J* = 1.8 Hz, 1H), 6.79–6.86 (m, 3H), 7.24–7.29 (m, 1H); ^13^C-NMR (CDCl_3_) δ 14.2 (CH_3_), 14.3 (CH_3_), 46.8 (CH_2_), 53.8 (CH), 55.2 (CH_3_), 59.5 (CH_2_), 100.6 (C) 105.8 (C), 110.7 (CH), 112.7 (CH), 116.7 (CH), 129.0 (C), 129.7 (CH), 131.8 (CH), 143.6 (C), 159.7 (C), 165.5 (C), 166.9 (C); IR (neat) 2981, 1702, 1644 cm^−1^; EI-MS *m*/*z* (rel intensity) 380 ([M + 2]^+^, 10), 378 ([M]^+^, 10), 332 (21), 253 (100); HRMS [M]^+^ calcd. for C_18_H_19_BrO_4_: 378.0467, found 378.0461.

*Ethyl 5-bromo-6a-(3-bromophenyl)-2-methyl-4,6a-dihydro-3aH-cyclopenta[b]furan-3-carboxylate* (**1g**). Yield 112 mg (87%). TLC (Et_2_O/hexanes (1:10)) *R*_f_ = 0.53; ^1^H-NMR (CDCl_3_) δ 1.24 (t, *J* = 7.2 Hz, 3H), 2.28 (d, *J* = 1.5 Hz, 3H), 2.83 (dt, *J* = 17.7, 2.1 Hz, 1H), 3.15 (ddd, *J* = 17.7, 7.8, 2.1 Hz, 1H), 3.70 (dt, *J* = 7.8, 2.1 Hz, 1H), 4.06–4.21 (m, 2H), 5.95 (t, *J* = 2.1 Hz, 1H), 7.15–7.20 (m, 2H), 7.37–7.41 (m, 2H); ^13^C-NMR (CDCl_3_) δ 14.2 (CH_3_), 14.3 (CH_3_), 46.7 (CH_2_), 53.9 (CH), 59.6 (CH_2_), 99.9 (C) 105.8 (C), 122.7 (C), 123.7 (CH), 127.6 (CH), 129.6 (CH), 130.1 (CH), 131.0 (CH), 131.3 (CH), 144.2 (C), 165.2 (C), 166.6 (C); IR (neat) 2979, 1704, 1643 cm^−1^; EI-MS *m*/*z* (rel intensity) 428 ([M + 2]^+^, 19), 426 ([M]^+^, 10), 382 (59), 301 (100); HRMS [M]^+^ calcd. for C_17_H_16_Br_2_O_3_: 425.9466, found 425.9460.

*Ethyl 5-bromo-2-methyl-6a-(naphthalen-2-yl)-4,6a-dihydro-3aH-cyclopenta[b]furan-3-carboxylate* (**1h**). Yield 77 mg (64%). TLC (Et_2_O/hexanes (1:10)) *R*_f_ = 0.48; ^1^H-NMR (CDCl_3_) δ 1.26 (t, *J* = 7.2 Hz, 3H), 2.35 (d, *J* = 1.5 Hz, 3H), 2.90 (dt, *J* = 17.4, 1.8 Hz, 1H), 3.22 (ddd, *J* = 17.4, 7.8, 1.8 Hz, 1H), 3.84 (dt, *J* = 7.8, 1.8 Hz, 1H), 4.11–4.23 (m, 2H), 6.09 (t, *J* = 1.8 Hz, 1H), 7.35 (dd, *J* = 8.4, 1.8 Hz, 1H), 7.44–7.51 (m, 2H), 7.74 (s, 1H), 7.80–7.85 (m, 3H); ^13^C-NMR (CDCl_3_) δ 14.3 (CH_3_), 14.4 (CH_3_), 46.9 (CH_2_), 53.7 (CH), 59.6 (CH_2_), 100.9 (C) 105.9 (C), 112.6 (CH), 123.1 (CH), 126.2 (CH), 126.4 (CH), 127.6 (CH), 128.1 (CH), 128.7 (CH), 129.3 (C), 131.8 (CH), 132.8 (C), 132.9 (C), 139.0 (C), 165.5 (C), 167.0 (C); IR (neat) 3058, 1700, 1637 cm^−1^; EI-MS *m*/*z* (rel intensity) 400 ([M + 2]^+^, 11), 398 ([M]^+^, 11), 273 (99), 202 (100); HRMS [M]+ calcd. for C_21_H_19_BrO_3_: 398.0518, found 398.0516.

#### 3.2.2. General Procedure for the Suzuki Reactions of Cyclopenta[*b*]furans **1**

To a solution of cyclopenta[*b*]furan **1** (0.5 mmol) in dioxane/H_2_O (5 mL/1 mL) was added Cs_2_CO_3_ (1.5 mmol, 489 mg), PhB(OH)_2_ (0.75 mmol, 91 mg), and Pd(dppf)Cl_2_ (0.05 mmol, 37 mg) at ambient temperature. The resulting mixture was vacuumed and purged with N_2_ for 3 times. The reaction was heated to 100 °C under N_2_ for 2 h. The reaction was then cooled room temperature and concentrated in a rotary evaporator. EtOAc (5 mL) and H_2_O (1 mL) were added and the mixture was transferred to a separatory funnel. The aqueous layer was back extracted with EtOAc (5 mL × 2). The combined organic layers were dried over Na_2_SO_4_, filtered, and concentrated in a rotary evaporator. The residue was purified by silica gel chromatography to give the title product. The following compounds were prepared using this method:

*Ethyl 2-methyl-5,6a-diphenyl-4,6a-dihydro-3aH-cyclopenta[b]furan-3-carboxylate* (**4a**). Yield 149 mg (86%). A yellow oil; TLC (EtOAc/hexanes (1:10)) *R*_f_ = 0.50; ^1^H-NMR (CDCl_3_) δ 1.29 (t, *J* = 7.2 Hz, 3H), 2.31 (s, 3H), 3.02 (dt, *J* = 17.1, 2.1 Hz, 1H), 3.29 (ddd, *J* = 17.1, 7.8, 2.1 Hz, 1H), 3.88 (dt, *J* = 7.8, 1.5 Hz, 1H), 4.11–4.26 (m, 2H), 6.23 (t, *J* = 2.1 Hz, 1H), 7.26–7.39 (m, 8H), 7.54 (d, *J* = 7.8 Hz, 2H); ^13^C-NMR (CDCl_3_) δ 14.4, 40.2, 52.8, 59.3, 102.3, 106.4, 124.6, 125.5, 126.4, 127.5, 128.4, 128.5, 134.7, 143.1, 146.5, 165.9, 166.6; IR (neat) 2977, 1698, 1644 cm^−1^; EI-MS *m*/*z* (rel intensity) 346 ([M]^+^, 12), 300 (100), 257 (30), 229 (47); HRMS [M]^+^ calcd. for C_23_H_22_O_3_: 346.1569, found 346.1561.

*Ethyl 6a-([1,1′-biphenyl]-4-yl)-2-methyl-5-phenyl-4,6a-dihydro-3aH-cyclopenta[b]furan-3-carboxylate* (**4b**). Yield 180 mg (85%). A colorless solid, m.p. 118–120 °C; TLC (EtOAc/hexanes (1:10)) *R*_f_ = 0.35; ^1^H-NMR (CDCl_3_) δ 1.28 (t, *J* = 7.2 Hz, 3H), 2.31 (s, 3H), 3.02 (dt, *J* = 17.1, 1.8 Hz, 1H), 3.31 (ddd, *J* = 17.1, 6.0, 2.1 Hz, 1H), 3.91 (dt, *J* = 6.0, 1.8 Hz, 1H), 4.10–4.25 (m, 2H), 6.25 (t, *J* = 1.8 Hz, 1H), 7.30–7.45 (m, 8H), 7.53–7.55 (m, 6H); ^13^C-NMR (CDCl_3_) δ 14.3, 40.1, 52.8, 59.3, 102.2, 106.4, 125.0, 125.4, 126.3, 126.9, 127.1, 127.2, 128.3, 128.4, 128.6, 134.6, 140.4, 140.5, 142.1, 146.5, 165.8, 166.5; IR (neat) 3031, 1700, 1637 cm^−1^; EI-MS *m*/*z* (rel intensity) 422 ([M]^+^, 10), 376 (100), 333 (13), 305 (23); HRMS [M]^+^ calcd. for C_29_H_26_O_3_: 422.1882, found 422.1878.

*Ethyl 6a-(4-chlorophenyl)-2-methyl-5-phenyl-4,6a-dihydro-3aH-cyclopenta[b]furan-3-carboxylate* (**4c**). Yield 164 mg (86%). A yellow oil; TLC (EtOAc/hexanes (1:10)) *R*_f_ = 0.43; ^1^H-NMR (CDCl_3_) δ 1.31 (t, *J* = 7.2 Hz, 3H), 2.32 (s, 3H), 3.04 (dt, *J* = 17.1, 2.1 Hz, 1H), 3.30 (ddd, *J* = 17.1, 7.8, 2.1 Hz, 1H), 3.84 (dt, *J* = 7.8, 1.8 Hz, 1H), 4.13–4.29 (m, 2H), 6.19 (t, *J* = 1.8 Hz, 1H), 7.27–7.40 (m, 7H), 7.54 (d, *J* = 7.8 Hz, 2H); ^13^C-NMR (CDCl_3_) δ 14.4, 40.1, 52.9, 59.4, 101.8, 106.4, 124.6, 125.0, 126.1, 128.4, 128.5, 128.6, 133.3, 134.5, 141.7, 146.9, 165.7, 166.4; IR (neat) 2979, 1698, 1643 cm^−1^; EI-MS *m*/*z* (rel intensity) 382 ([M + 2]^+^, 4), 380 ([M]^+^, 10), 334 (100), 228 (36); HRMS [M]^+^ calcd. for C_23_H_21_ClO_3_: 380.1179, found 380.1184.

*Ethyl 2-methyl-5-phenyl-6a-(p-tolyl)-4,6a-dihydro-3aH-cyclopenta[b]furan-3-carboxylate* (**4d**). Yield 151 mg (84%). A yellow oil; TLC (EtOAc/hexanes (1:10)) *R*_f_ = 0.60; ^1^H-NMR (CDCl_3_) δ 1.31 (t, *J* = 7.2 Hz, 3H), 2.32 (s, 3H), 2.36 (s, 3H), 3.03 (dt, *J* = 17.1, 2.1 Hz, 1H), 3.30 (ddd, *J* = 17.1, 7.8, 2.1 Hz, 1H), 3.88 (dt, *J* = 7.8, 1.8 Hz, 1H), 4.17–4.25 (m, 2H), 6.24 (t, *J* = 1.8 Hz, 1H), 7.18 (d, *J* = 8.1 Hz, 2H), 7.25–7.40 (m, 5H), 7.56 (d, *J* = 8.1 Hz, 2H); ^13^C-NMR (CDCl_3_) δ 14.4, 14.5, 21.0, 40.2, 52.8, 59.3, 102.4, 106.4, 124.6, 125.6, 126.4, 128.4, 128.5, 129.1, 134.8, 137.3, 140.2, 146.3, 166.0, 166.6; IR (neat) 2977, 2923, 1693 cm^−1^; EI-MS *m*/*z* (rel intensity) 360 ([M]^+^, 11), 314 (100), 243 (27), 228 (20); HRMS [M]^+^ calcd. for C_24_H_24_O_3_: 360.1725, found 360.1721.

*Ethyl 6a-(4-methoxyphenyl)-2-methyl-5-phenyl-4,6a-dihydro-3aH-cyclopenta[b]furan-3-carboxylate* (**4e**). Yield 154 mg (82%). A red oil; TLC (EtOAc/hexanes (1:10)) *R*_f_ = 0.50; ^1^H-NMR (CDCl_3_) δ 1.29 (t, *J* = 7.2 Hz, 3H), 2.29 (s, 3H), 2.99 (dt, *J* = 17.1, 1.8 Hz, 1H), 3.26 (ddd, *J* = 17.1, 6.6, 2.1 Hz, 1H), 3.79 (s, 3H), 3.83 (dt, *J* = 6.6, 1.8 Hz, 1H), 4.13–4.24 (m, 2H), 6.22 (t, *J* = 1.8 Hz, 1H), 6.88 (d, *J* = 6.6 Hz, 2H), 7.25–7.38 (m, 5H), 7.53 (d, *J* = 7.8 Hz, 2H); ^13^C-NMR (CDCl_3_) δ 14.4, 14.5, 40.1, 52.7, 55.2, 59.4, 102.2, 106.4, 113.7, 125.5, 125.9, 126.4, 128.4, 128.5, 134.8, 135.3, 146.3, 159.0, 166.0, 166.6; IR (neat) 2931, 1696, 1644 cm^−1^; EI-MS *m*/*z* (rel intensity) 376 ([M]^+^, 18), 330 (100), 287 (19), 259 (33); HRMS [M]^+^ calcd. for C_24_H_24_O_4_: 376.1675, found 376.1672.

*Ethyl 6a-(3-methoxyphenyl)-2-methyl-5-phenyl-4,6a-dihydro-3aH-cyclopenta[b]furan-3-carboxylate* (**4f**). Yield 152 mg (81%). A yellow oil; TLC (EtOAc/hexanes (1:10)) *R*_f_ = 0.44; ^1^H-NMR (CDCl_3_) δ 1.30 (t, *J* = 7.2 Hz, 3H), 2.32 (s, 3H), 3.02 (dt, *J* = 17.1, 2.1 Hz, 1H), 3.30 (ddd, *J* = 17.1, 7.8, 2.1 Hz, 1H), 3.80 (s, 3H), 3.88 (dt, *J* = 7.8, 1.8 Hz, 1H), 4.16–4.24 (m, 2H), 6.22 (t, *J* = 1.8 Hz, 1H), 6.80–6.84 (m, 1H), 6.92–6.95 (m, 2H), 7.25–7.39 (m, 4H), 7.55 (d, *J* = 8.1 Hz, 2H); ^13^C-NMR (CDCl_3_) δ 14.4, 40.2, 52.8, 55.2, 59.4, 102.2, 106.5, 110.7, 112.4, 116.9, 125.4, 126.4, 128.4, 128.5, 129.5, 134.7, 144.8, 146.5, 159.7, 165.9, 166.5; IR (neat) 3056, 2979, 1693 cm^−1^; EI-MS *m*/*z* (rel intensity) 376 ([M]^+^, 18), 330 (100), 287 (48), 259 (41); HRMS [M]^+^ calcd. for C_24_H_24_O_4_: 376.1675, found 376.1680.

*Ethyl 6a-([1,1′-biphenyl]-3-yl)-2-methyl-5-phenyl-4,6a-dihydro-3aH-cyclopenta[b]furan-3-carboxylate* (**4g**). Yield 180 mg (85%). A red oil; TLC (EtOAc/hexanes (1:10)) *R*_f_ = 0.53; ^1^H-NMR (CDCl_3_) δ 1.33 (t, *J* = 7.2 Hz, 3H), 2.38 (s, 3H), 3.10 (dt, *J* = 17.1, 1.8 Hz, 1H), 3.38 (ddd, *J* = 17.1, 6.0, 2.1 Hz, 1H), 3.99 (dt, *J* = 6.0, 1.8 Hz, 1H), 4.20–4.28 (m, 2H), 6.32 (t, *J* = 1.8 Hz, 1H), 7.34–7.49 (m, 8H), 7.53–7.63 (m, 6H); ^13^C-NMR (CDCl_3_) δ 14.4, 14.5, 40.3, 52.9, 59.4, 102.4, 106.5, 123.4, 123.6, 125.5, 126.4, 126.5, 127.2, 127.3, 128.4, 128.5, 128.7, 128.9, 134.7, 141.0, 141.5, 143.7, 146.6, 165.9, 166.6; IR (neat) 3060, 2981, 1706 cm^−1^; EI-MS *m*/*z* (rel intensity) 422 ([M]^+^, 13), 376 (100), 333 (23), 305 (27); HRMS [M]^+^ calcd. for C_29_H_26_O_3_: 422.1882, found 422.1884.

*Ethyl 2-methyl-6a-(naphthalen-2-yl)-5-phenyl-4,6a-dihydro-3aH-cyclopenta[b]furan-3-carboxylate* (**4h**). Yield 167 mg (84%). A yellow oil; TLC (EtOAc/hexanes (1:10)) *R*_f_ = 0.38; ^1^H-NMR (MHz, CDCl_3_) δ 1.31 (t, *J* = 7.2 Hz, 3H), 2.38 (s, 3H), 3.09 (dt, *J* = 17.1, 1.8 Hz, 1H), 3.37 (ddd, *J* = 17.1, 7.8, 1.8 Hz, 1H), 3.98 (d, *J* = 7.8 Hz, 1H), 4.14–4.29 (m, 2H), 6.34 (t, *J* = 1.8 Hz, 1H), 7.33–7.49 (m, 6H), 7.58–7.61 (m, 2H), 7.75–7.85 (m, 4H); ^13^C-NMR (CDCl_3_) δ 14.5, 14.6, 40.4, 52.9, 59.5, 102.6, 106.7, 123.2, 125.5, 126.1, 126.3, 126.5, 127.6, 128.2, 128.5, 128.6, 132.8, 133.1, 134.8, 140.4, 146.9, 166.1, 166.8; IR (neat) 3056, 2977, 1698 cm^−1^; EI-MS *m*/*z* (rel intensity) 396 ([M]^+^, 22), 350 (100), 307 (23), 279 (53); HRMS [M]^+^ calcd. for C_27_H_24_O_3_: 396.1725, found 396.1733.

## 4. Conclusions 

In summary, the synthetic application of hypervalent iodine(III) in the formation of cyclopenta[*b*]furans is described. This method provides an alternative route to access the target for those cases where the corresponding diene-aldehyde precursor is not readily available or its use limited by intrinsic potential instability.

## Figures and Tables

**Table 1:**
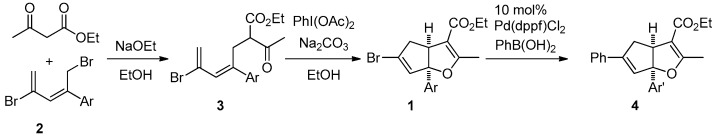
Synthesis of cyclopenta[*b*]furans.

Entry	Ar	3, Yield (%)	1, Yield (%) ^a^	4, Yield (%) ^c^
1	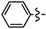	--	**1a**, 72; (73) ^b^	**4a**, Ar’ = Ar, 86
2	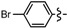	--	**1b**, 78; (71) ^b^	**4b**, Ar’ = 4-PhC_6_H_4_, 85
3	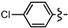	--	**1c**, 90; (70) ^b^	**4c**, Ar’ = Ar, 86
4	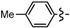	--	**1d**, 70	**4d**, Ar’ = Ar, 84
5	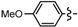	--	**1e**, 65	**4e**, Ar’ = Ar, 82
6	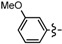	--	**1f**, 63	**4f**, Ar’ = Ar, 81
7	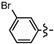	--	**1g**, 87	**4g**, Ar’ = 3-PhC_6_H_4_, 85
8	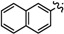	**3g**, 58	**1h**, 64	**4h**, Ar’ = Ar, 84

^a^ *Reagents and Conditions*: **3** (0.3 mmol), PhI(OAc)_2_ (0.6 mmol) and Na_2_CO_3_ (0.66 mmol) in EtOH (1.2 mL). ^b^ PhI(OCOCF_3_)_2_ was employed instead of PhI(OAc)_2_. ^c^ **1** (0.5 mmol), Cs_2_CO_3_ (1.5 mmol, 3.0 mmol for **4b**, **4g**), PhB(OH)_2_ (0.75 mmol, 1.5 mmol for **4b**, **4g**), Pd(dppf)Cl_2_ (0.05 mmol) in dioxane/water (5/1, 0.5 mL) 100 °C for 2 h.

**Table 2:**
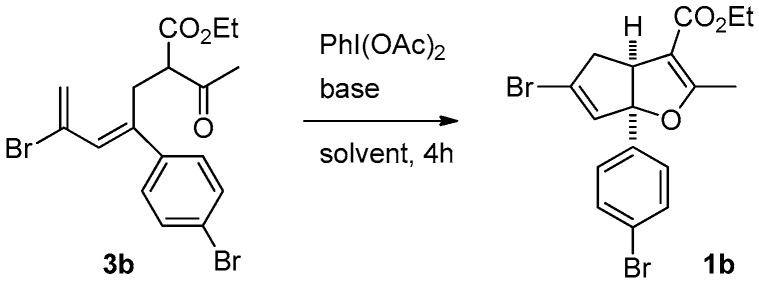
Reaction Optimizations ^a^.

Entry	Base	Solvent, Temp	Yield (%) ^a^
1	NaHCO_3_	CH_3_CN, r.t.	0
2	NaHCO_3_	CH_3_CN, reflux	48
3	Na_2_CO_3_	CH_3_CN, reflux	60
4	K_2_CO_3_	CH_3_CN, reflux	53
5	DBU	CH_3_CN, reflux	12
6	Na_2_CO_3_	PhCH_3_, reflux	26
7	Na_2_CO_3_	EtOH, reflux	78 (71) ^b^
8	Na_2_CO_3_	EtOH, r.t.	0
9	DMAP	EtOH, reflux	31

^a^ *Reagents and Conditions*: **3b** (0.3 mmol), PhI(OAc)_2_ (0.6 mmol) and base (0.66 mmol) in solvent (1.2 mL). ^b^ **3b** (0.3 mmol), PhI(OCOCF_3_)_2_ (0.6 mmol) and Na_2_CO_3_ (0.66 mmol) in EtOH (1.2 mL) reflux.

## Supplementary Materials

Supplementary materials can be accessed at: http://www.mdpi.com/1420-3049/21/12/1713/s1.
