# Supplementary material for: Hypervalent Iodine(III)-Induced Domino Oxidative Cyclization for the Synthesis of Cyclopenta[b]furans
Source: Molecules. 2016 Dec 21;21(12):1713. doi: 10.3390/molecules21121713 (PMC6274509; doi:10.3390/molecules21121713)
Supplement: Supplementary file 1 [file molecules-21-01713-s001.pdf]

Supplementary Materials: Hypervalent Iodine(III)-Induced Domino Oxidative Cyclization for Synthesis of Cyclopenta[*b*]furans

Mei-Huey Lin, Yu-Chun Chen, Shih-Hao Chiu, Yun-Fan Chen and Tsung-Hsun Chuang

I. X-ray Crystallographic Analyses

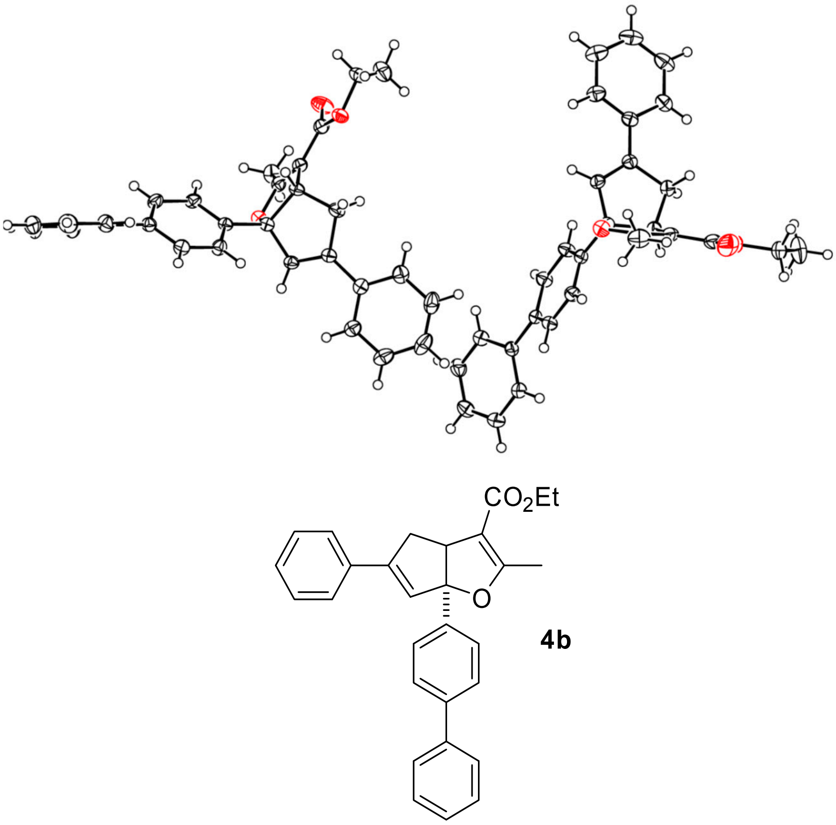

Figure S1. ORTEP plot of x-ray crystallographic data for **4b**.

Table S1. Crystal data and structure refinement for **4b** (CCDC: 1517150, cyc43).

Entry	Data	Data
Identification code	cyc43	
Empirical formula	$C_{58}{\cdot}H_{52}{\cdot}O_6$	
Formula weight	845 g/mol	
Temperature	150 K	
Wavelength	0.71073 Å	
Crystal system	Monoclinic	
Space group	P2(1)/c	
	a = 23.1962(5) Å	α = 90°.
Unit cell dimensions	*b* = 10.0132(2) Å	β = 110.2600(10)°
	c = 20.5387(5) Å	γ = 90°
Volume	4475.34(17) $Å^3$	
Z	4	
Density (calculated)	1.254 Mg/m^3	
Absorption coefficient	0.080 mm^{-1}	
F(000)	1792	

Crystal size	0.12 × 0.12 × 0.12 mm^3
Theta range for data collection	0.94° to 28.75°.
Index ranges	−31 ≤ h ≤ 31, −13 ≤ k ≤ 13, −27 ≤ l ≤ 25
Reflections collected	42,416
Independent reflections	11,583 [R(int) = 0.0324]
Completeness to theta = 28.75°	99.70%
Max. and min. transmission	0.9905 and 0.9905
Refinement method	Full-matrix least-squares on F2
Data/restraints/parameters	11,583/0/581
Goodness-of-fit on F2	1.026
Final R indices [I > 2sigma(I)]	R1 = 0.0427, wR2 = 0.1040
R indices (all data)	R1 = 0.0584, wR2 = 0.1147
Largest diff. peak and hole	0.288 and −0.232 e.Å^{-3}

Table S2. Atomic coordinates (×10^4) and equivalent isotropic displacement parameters (Å^2 × 10^3) for cyc43. U(eq) is defined as one third of the trace of the orthogonalized U^{ij} tensor.

Atom	*x*	*y*	*z*	U(eq)
O(1)	−1490(1)	4296(1)	7126(1)	36(1)
O(2)	−1895(1)	5865(1)	6309(1)	50(1)
O(3)	19(1)	6544(1)	6651(1)	29(1)
O(4)	4387(1)	9050(1)	8659(1)	27(1)
O(5)	5121(1)	7968(1)	950s7(1)	40(1)
O(6)	5532(1)	6382(1)	7783(1)	23(1)
C(1)	−2058(1)	2825(2)	7584(1)	70(1)
C(2)	−2110(1)	3907(2)	7066(1)	47(1)
C(3)	−1448(1)	5314(1)	6711(1)	33(1)
C(4)	−805(1)	5613(1)	6827(1)	28(1)
C(5)	−273(1)	4926(1)	7360(1)	24(1)
C(6)	285(1)	5592(1)	7228(1)	23(1)
C(7)	696(1)	4634(1)	7019(1)	23(1)
C(8)	1185(1)	4019(1)	7526(1)	28(1)
C(9)	1552(1)	3090(1)	7355(1)	27(1)
C(10)	1442(1)	2748(1)	6664(1)	23(1)
C(11)	1832(1)	1747(1)	6478(1)	24(1)
C(12)	2454(1)	1623(1)	6868(1)	29(1)
C(13)	2821(1)	703(1)	6689(1)	34(1)
C(14)	2571(1)	−106(2)	6114(1)	36(1)
C(15)	−197(1)	5240(1)	8121(1)	26(1)
C(16)	354(1)	6156(1)	8369(1)	23(1)
C(17)	566(1)	6761(1)	9066(1)	25(1)
C(18)	1016(1)	7753(1)	9242(1)	33(1)
C(19)	1197(1)	8368(2)	9883(1)	42(1)
C(20)	936(1)	7996(2)	10,370(1)	43(1)
C(21)	497(1)	7006(2)	10,209(1)	40(1)
C(22)	311(1)	6393(1)	9563(1)	32(1)
C(23)	604(1)	6343(1)	7881(1)	25(1)
C(24)	582(1)	4293(1)	6329(1)	26(1)
C(25)	953(1)	3371(1)	6158(1)	26(1)
C(26)	1586(1)	913(1)	5903(1)	30(1)
C(27)	1953(1)	−1(1)	5720(1)	36(1)
C(28)	−604(1)	6495(1)	6465(1)	29(1)
C(29)	−940(1)	7408(2)	5890(1)	41(1)

C(30)	2502(1)	4062(2)	5608(1)	42(1)
C(31)	3039(1)	3941(2)	5459(1)	38(1)
C(32)	3547(1)	4711(1)	5812(1)	29(1)
C(33)	3526(1)	5639(1)	6313(1)	23(1)
C(34)	4070(1)	6413(1)	6726(1)	22(1)
C(35)	4641(1)	6262(1)	6727(1)	22(1)
C(36)	5095(1)	7180(1)	7218(1)	20(1)
C(37)	5475(1)	8004(1)	6897(1)	21(1)
C(38)	5243(1)	8327(1)	6194(1)	25(1)
C(39)	5584(1)	9084(1)	5893(1)	26(1)
C(40)	6170(1)	9533(1)	6283(1)	23(1)
C(41)	6541(1)	10,332(1)	5961(1)	25(1)
C(42)	6268(1)	11,336(1)	5485(1)	31(1)
C(43)	6611(1)	12,090(1)	5184(1)	38(1)
C(44)	7231(1)	11,856(2)	5354(1)	40(1)
C(45)	3660(1)	10,553(2)	8833(1)	45(1)
C(46)	4242(1)	9809(1)	9185(1)	33(1)
C(47)	4860(1)	8184(1)	8894(1)	25(1)
C(48)	5010(1)	7608(1)	8321(1)	22(1)
C(49)	4704(1)	7984(1)	7569(1)	21(1)
C(50)	2471(1)	4980(2)	6093(1)	40(1)
C(51)	2973(1)	5779(1)	6434(1)	31(1)
C(52)	4038(1)	7484(1)	7231(1)	26(1)
C(53)	5455(1)	6706(1)	8388(1)	23(1)
C(54)	5883(1)	6004(1)	9003(1)	32(1)
C(55)	7507(1)	10,868(2)	5820(1)	46(1)
C(56)	7166(1)	10,110(2)	6123(1)	40(1)
C(57)	6401(1)	9205(1)	6986(1)	26(1)
C(58)	6058(1)	8461(1)	7289(1)	25(1)

Table S3. Bond lengths (Å) and angles (°) for cyc43.

Bond	Length (Angle)
O(1)-C(3)	1.3529(18)
O(1)-C(2)	1.4526(16)
O(2)-C(3)	1.2106(17)
O(3)-C(28)	1.3625(16)
O(3)-C(6)	1.4791(14)
O(4)-C(47)	1.3500(15)
O(4)-C(46)	1.4520(14)
O(5)-C(47)	1.2126(15)
O(6)-C(53)	1.3566(14)
O(6)-C(36)	1.4831(13)
C(1)-C(2)	1.494(3)
C(1)-H(1A)	0.96
C(1)-H(1B)	0.96
C(1)-H(1C)	0.96
C(2)-H(2A)	0.97
C(2)-H(2B)	0.97
C(3)-C(4)	1.4588(18)
C(4)-C(28)	1.3375(19)
C(4)-C(5)	1.5036(17)
C(5)-C(15)	1.5438(17)

C(5)-C(6)	1.5619(16)
C(5)-H(5)	0.98
C(6)-C(23)	1.4917(16)
C(6)-C(7)	1.5150(16)
C(7)-C(24)	1.3898(16)
C(7)-C(8)	1.3910(17)
C(8)-C(9)	1.3859(17)
C(8)-H(8)	0.93
C(9)-C(10)	1.3953(16)
C(9)-H(9)	0.93
C(10)-C(25)	1.3919(17)
C(10)-C(11)	1.4863(16)
C(11)-C(12)	1.3924(17)
C(11)-C(26)	1.3960(17)
C(12)-C(13)	1.3865(18)
C(12)-H(12)	0.93
C(13)-C(14)	1.383(2)
C(13)-H(13)	0.93
C(14)-C(27)	1.386(2)
C(14)-H(14)	0.93
C(15)-C(16)	1.5108(16)
C(15)-H(15A)	0.97
C(15)-H(15B)	0.97
C(16)-C(23)	1.3321(17)
C(16)-C(17)	1.4747(16)
C(17)-C(22)	1.3931(18)
C(17)-C(18)	1.3951(18)
C(18)-C(19)	1.3812(19)
C(18)-H(18)	0.93
C(19)-C(20)	1.385(2)
C(19)-H(19)	0.93
C(20)-C(21)	1.378(2)
C(20)-H(20)	0.93
C(21)-C(22)	1.3886(19)
C(21)-H(21)	0.93
C(22)-H(22)	0.93
C(23)-H(23)	0.93
C(24)-C(25)	1.3879(17)
C(24)-H(24)	0.93
C(25)-H(25)	0.93
C(26)-C(27)	1.3865(18)
C(26)-H(26)	0.93
C(27)-H(27)	0.93
C(28)-C(29)	1.4834(18)
C(29)-H(29A)	0.96
C(29)-H(29B)	0.96
C(29)-H(29C)	0.96
C(30)-C(50)	1.376(2)
C(30)-C(31)	1.386(2)
C(30)-H(30)	0.93
C(31)-C(32)	1.3848(18)
C(31)-H(31)	0.93

C(32)-C(33)	1.4007(17)
C(32)-H(32)	0.93
C(33)-C(51)	1.3959(17)
C(33)-C(34)	1.4740(16)
C(34)-C(35)	1.3316(16)
C(34)-C(52)	1.5111(16)
C(35)-C(36)	1.4943(16)
C(35)-H(35)	0.93
C(36)-C(37)	1.5152(16)
C(36)-C(49)	1.5626(15)
C(37)-C(58)	1.3897(16)
C(37)-C(38)	1.3937(16)
C(38)-C(39)	1.3855(17)
C(38)-H(38)	0.93
C(39)-C(40)	1.3915(17)
C(39)-H(39)	0.93
C(40)-C(57)	1.3942(16)
C(40)-C(41)	1.4882(16)
C(41)-C(56)	1.3886(18)
C(41)-C(42)	1.3915(17)
C(42)-C(43)	1.3881(18)
C(42)-H(42)	0.93
C(43)-C(44)	1.377(2)
C(43)-H(43)	0.93
C(44)-C(55)	1.372(2)
C(44)-H(44)	0.93
C(45)-C(46)	1.491(2)
C(45)-H(45A)	0.96
C(45)-H(45B)	0.96
C(45)-H(45C)	0.96
C(46)-H(46A)	0.97
C(46)-H(46B)	0.97
C(47)-C(48)	1.4574(16)
C(48)-C(53)	1.3422(17)
C(48)-C(49)	1.5072(15)
C(49)-C(52)	1.5415(17)
C(49)-H(49)	0.98
C(50)-C(51)	1.3863(19)
C(50)-H(50)	0.93
C(51)-H(51)	0.93
C(52)-H(52A)	0.97
C(52)-H(52B)	0.97
C(53)-C(54)	1.4852(16)
C(54)-H(54A)	0.96
C(54)-H(54B)	0.96
C(54)-H(54C)	0.96
C(55)-C(56)	1.387(2)
C(55)-H(55)	0.93
C(56)-H(56)	0.93
C(57)-C(58)	1.3859(17)
C(57)-H(57)	0.93
C(58)-H(58)	0.93

C(3)-O(1)-C(2)	115.64(12)
C(28)-O(3)-C(6)	108.05(9)
C(47)-O(4)-C(46)	115.99(10)
C(53)-O(6)-C(36)	108.43(8)
C(2)-C(1)-H(1A)	109.5
C(2)-C(1)-H(1B)	109.5
H(1A)-C(1)-H(1B)	109.5
C(2)-C(1)-H(1C)	109.5
H(1A)-C(1)-H(1C)	109.5
H(1B)-C(1)-H(1C)	109.5
O(1)-C(2)-C(1)	107.53(14)
O(1)-C(2)-H(2A)	110.2
C(1)-C(2)-H(2A)	110.2
O(1)-C(2)-H(2B)	110.2
C(1)-C(2)-H(2B)	110.2
H(2A)-C(2)-H(2B)	108.5
O(2)-C(3)-O(1)	122.86(13)
O(2)-C(3)-C(4)	126.98(14)
O(1)-C(3)-C(4)	110.16(11)
C(28)-C(4)-C(3)	125.40(12)
C(28)-C(4)-C(5)	110.54(11)
C(3)-C(4)-C(5)	124.02(12)
C(4)-C(5)-C(15)	114.92(10)
C(4)-C(5)-C(6)	101.47(10)
C(15)-C(5)-C(6)	106.11(9)
C(4)-C(5)-H(5)	111.3
C(15)-C(5)-H(5)	111.3
C(6)-C(5)-H(5)	111.3
O(3)-C(6)-C(23)	109.58(9)
O(3)-C(6)-C(7)	108.05(9)
C(23)-C(6)-C(7)	114.22(10)
O(3)-C(6)-C(5)	105.84(9)
C(23)-C(6)-C(5)	103.98(9)
C(7)-C(6)-C(5)	114.79(10)
C(24)-C(7)-C(8)	118.24(11)
C(24)-C(7)-C(6)	121.85(11)
C(8)-C(7)-C(6)	119.83(10)
C(9)-C(8)-C(7)	121.31(11)
C(9)-C(8)-H(8)	119.3
C(7)-C(8)-H(8)	119.3
C(8)-C(9)-C(10)	120.77(12)
C(8)-C(9)-H(9)	119.6
C(10)-C(9)-H(9)	119.6
C(25)-C(10)-C(9)	117.59(11)
C(25)-C(10)-C(11)	121.53(11)
C(9)-C(10)-C(11)	120.88(11)
C(12)-C(11)-C(26)	118.13(11)
C(12)-C(11)-C(10)	120.99(11)
C(26)-C(11)-C(10)	120.88(11)
C(13)-C(12)-C(11)	121.01(12)
C(13)-C(12)-H(12)	119.5
C(11)-C(12)-H(12)	119.5

C(14)-C(13)-C(12)	120.11(12)
C(14)-C(13)-H(13)	119.9
C(12)-C(13)-H(13)	119.9
C(13)-C(14)-C(27)	119.78(12)
C(13)-C(14)-H(14)	120.1
C(27)-C(14)-H(14)	120.1
C(16)-C(15)-C(5)	104.56(9)
C(16)-C(15)-H(15A)	110.8
C(5)-C(15)-H(15A)	110.8
C(16)-C(15)-H(15B)	110.8
C(5)-C(15)-H(15B)	110.8
H(15A)-C(15)-H(15B)	108.9
C(23)-C(16)-C(17)	125.96(11)
C(23)-C(16)-C(15)	111.71(10)
C(17)-C(16)-C(15)	122.33(10)
C(22)-C(17)-C(18)	117.90(12)
C(22)-C(17)-C(16)	121.31(12)
C(18)-C(17)-C(16)	120.76(11)
C(19)-C(18)-C(17)	121.17(13)
C(19)-C(18)-H(18)	119.4
C(17)-C(18)-H(18)	119.4
C(18)-C(19)-C(20)	120.20(14)
C(18)-C(19)-H(19)	119.9
C(20)-C(19)-H(19)	119.9
C(21)-C(20)-C(19)	119.48(13)
C(21)-C(20)-H(20)	120.3
C(19)-C(20)-H(20)	120.3
C(20)-C(21)-C(22)	120.38(14)
C(20)-C(21)-H(21)	119.8
C(22)-C(21)-H(21)	119.8
C(21)-C(22)-C(17)	120.85(13)
C(21)-C(22)-H(22)	119.6
C(17)-C(22)-H(22)	119.6
C(16)-C(23)-C(6)	113.33(11)
C(16)-C(23)-H(23)	123.3
C(6)-C(23)-H(23)	123.3
C(25)-C(24)-C(7)	120.35(11)
C(25)-C(24)-H(24)	119.8
C(7)-C(24)-H(24)	119.8
C(24)-C(25)-C(10)	121.75(11)
C(24)-C(25)-H(25)	119.1
C(10)-C(25)-H(25)	119.1
C(27)-C(26)-C(11)	120.99(12)
C(27)-C(26)-H(26)	119.5
C(11)-C(26)-H(26)	119.5
C(14)-C(27)-C(26)	119.98(13)
C(14)-C(27)-H(27)	120
C(26)-C(27)-H(27)	120
C(4)-C(28)-O(3)	114.07(11)
C(4)-C(28)-C(29)	131.41(13)
O(3)-C(28)-C(29)	114.50(12)
C(28)-C(29)-H(29A)	109.5

C(28)-C(29)-H(29B)	109.5
H(29A)-C(29)-H(29B)	109.5
C(28)-C(29)-H(29C)	109.5
H(29A)-C(29)-H(29C)	109.5
H(29B)-C(29)-H(29C)	109.5
C(50)-C(30)-C(31)	119.66(13)
C(50)-C(30)-H(30)	120.2
C(31)-C(30)-H(30)	120.2
C(32)-C(31)-C(30)	120.25(14)
C(32)-C(31)-H(31)	119.9
C(30)-C(31)-H(31)	119.9
C(31)-C(32)-C(33)	120.84(13)
C(31)-C(32)-H(32)	119.6
C(33)-C(32)-H(32)	119.6
C(51)-C(33)-C(32)	117.81(11)
C(51)-C(33)-C(34)	120.10(11)
C(32)-C(33)-C(34)	122.04(11)
C(35)-C(34)-C(33)	126.45(11)
C(35)-C(34)-C(52)	111.41(10)
C(33)-C(34)-C(52)	122.12(10)
C(34)-C(35)-C(36)	113.50(10)
C(34)-C(35)-H(35)	123.3
C(36)-C(35)-H(35)	123.3
O(6)-C(36)-C(35)	109.17(9)
O(6)-C(36)-C(37)	106.81(9)
C(35)-C(36)-C(37)	114.91(9)
O(6)-C(36)-C(49)	105.44(8)
C(35)-C(36)-C(49)	104.03(9)
C(37)-C(36)-C(49)	116.00(9)
C(58)-C(37)-C(38)	118.21(11)
C(58)-C(37)-C(36)	121.55(10)
C(38)-C(37)-C(36)	120.24(10)
C(39)-C(38)-C(37)	120.92(11)
C(39)-C(38)-H(38)	119.5
C(37)-C(38)-H(38)	119.5
C(38)-C(39)-C(40)	121.03(11)
C(38)-C(39)-H(39)	119.5
C(40)-C(39)-H(39)	119.5
C(39)-C(40)-C(57)	117.87(11)
C(39)-C(40)-C(41)	121.22(10)
C(57)-C(40)-C(41)	120.91(11)
C(56)-C(41)-C(42)	117.96(12)
C(56)-C(41)-C(40)	121.58(11)
C(42)-C(41)-C(40)	120.45(11)
C(43)-C(42)-C(41)	120.84(13)
C(43)-C(42)-H(42)	119.6
C(41)-C(42)-H(42)	119.6
C(44)-C(43)-C(42)	120.30(13)
C(44)-C(43)-H(43)	119.9
C(42)-C(43)-H(43)	119.9
C(55)-C(44)-C(43)	119.55(13)
C(55)-C(44)-H(44)	120.2

C(43)-C(44)-H(44)	120.2
C(46)-C(45)-H(45A)	109.5
C(46)-C(45)-H(45B)	109.5
H(45A)-C(45)-H(45B)	109.5
C(46)-C(45)-H(45C)	109.5
H(45A)-C(45)-H(45C)	109.5
H(45B)-C(45)-H(45C)	109.5
O(4)-C(46)-C(45)	107.77(11)
O(4)-C(46)-H(46A)	110.2
C(45)-C(46)-H(46A)	110.2
O(4)-C(46)-H(46B)	110.2
C(45)-C(46)-H(46B)	110.2
H(46A)-C(46)-H(46B)	108.5
O(5)-C(47)-O(4)	122.57(11)
O(5)-C(47)-C(48)	126.57(12)
O(4)-C(47)-C(48)	110.84(10)
C(53)-C(48)-C(47)	124.90(11)
C(53)-C(48)-C(49)	110.28(10)
C(47)-C(48)-C(49)	124.77(10)
C(48)-C(49)-C(52)	115.01(10)
C(48)-C(49)-C(36)	101.62(9)
C(52)-C(49)-C(36)	106.01(9)
C(48)-C(49)-H(49)	111.2
C(52)-C(49)-H(49)	111.2
C(36)-C(49)-H(49)	111.2
C(30)-C(50)-C(51)	120.38(13)
C(30)-C(50)-H(50)	119.8
C(51)-C(50)-H(50)	119.8
C(50)-C(51)-C(33)	120.99(13)
C(50)-C(51)-H(51)	119.5
C(33)-C(51)-H(51)	119.5
C(34)-C(52)-C(49)	105.02(9)
C(34)-C(52)-H(52A)	110.7
C(49)-C(52)-H(52A)	110.7
C(34)-C(52)-H(52B)	110.7
C(49)-C(52)-H(52B)	110.7
H(52A)-C(52)-H(52B)	108.8
C(48)-C(53)-O(6)	114.04(10)
C(48)-C(53)-C(54)	132.12(11)
O(6)-C(53)-C(54)	113.83(10)
C(53)-C(54)-H(54A)	109.5
C(53)-C(54)-H(54B)	109.5
H(54A)-C(54)-H(54B)	109.5
C(53)-C(54)-H(54C)	109.5
H(54A)-C(54)-H(54C)	109.5
H(54B)-C(54)-H(54C)	109.5
C(44)-C(55)-C(56)	120.46(14)
C(44)-C(55)-H(55)	119.8
C(56)-C(55)-H(55)	119.8
C(55)-C(56)-C(41)	120.89(14)
C(55)-C(56)-H(56)	119.6
C(41)-C(56)-H(56)	119.6

C(58)-C(57)-C(40)	121.20(11)
C(58)-C(57)-H(57)	119.4
C(40)-C(57)-H(57)	119.4
C(57)-C(58)-C(37)	120.77(11)
C(57)-C(58)-H(58)	119.6
C(37)-C(58)-H(58)	119.6

Table S4. Anisotropic displacement parameters ($Å^2 \times 10^3$) for cyc43. The anisotropic displacement factor exponent takes the form: $-2\pi^2[h^2 a^{*2}U^{11} + ... + 2 h k a^* b^* U^{12}]$.

Atom	U11	U22	U33	U23	U13	U12
O(1)	24(1)	43(1)	42(1)	−8(1)	14(1)	−3(1)
O(2)	30(1)	61(1)	48(1)	−2(1)	−2(1)	4(1)
O(3)	32(1)	26(1)	28(1)	3(1)	11(1)	3(1)
O(4)	32(1)	28(1)	23(1)	−3(1)	12(1)	0(1)
O(5)	51(1)	48(1)	20(1)	1(1)	11(1)	11(1)
O(6)	25(1)	22(1)	22(1)	4(1)	8(1)	4(1)
C(1)	57(1)	88(2)	82(1)	−4(1)	47(1)	−19(1)
C(2)	26(1)	62(1)	57(1)	−23(1)	20(1)	−9(1)
C(3)	28(1)	38(1)	30(1)	−11(1)	5(1)	1(1)
C(4)	26(1)	29(1)	25(1)	−6(1)	5(1)	1(1)
C(5)	22(1)	22(1)	28(1)	−2(1)	10(1)	−1(1)
C(6)	26(1)	21(1)	24(1)	1(1)	10(1)	1(1)
C(7)	24(1)	22(1)	26(1)	−2(1)	11(1)	−3(1)
C(8)	32(1)	30(1)	21(1)	−3(1)	10(1)	2(1)
C(9)	28(1)	28(1)	25(1)	0(1)	9(1)	3(1)
C(10)	23(1)	22(1)	26(1)	−4(1)	12(1)	−5(1)
C(11)	26(1)	24(1)	28(1)	−2(1)	14(1)	−2(1)
C(12)	29(1)	31(1)	30(1)	−5(1)	12(1)	−2(1)
C(13)	26(1)	40(1)	38(1)	−2(1)	13(1)	4(1)
C(14)	36(1)	36(1)	44(1)	−7(1)	21(1)	4(1)
C(15)	23(1)	28(1)	26(1)	2(1)	9(1)	0(1)
C(16)	24(1)	19(1)	26(1)	0(1)	8(1)	4(1)
C(17)	26(1)	23(1)	25(1)	1(1)	8(1)	6(1)
C(18)	40(1)	29(1)	27(1)	−1(1)	9(1)	−2(1)
C(19)	51(1)	34(1)	33(1)	−6(1)	6(1)	−5(1)
C(20)	54(1)	42(1)	28(1)	−9(1)	8(1)	10(1)
C(21)	45(1)	50(1)	30(1)	−1(1)	18(1)	10(1)
C(22)	31(1)	36(1)	31(1)	0(1)	14(1)	4(1)
C(23)	25(1)	20(1)	30(1)	−3(1)	10(1)	−2(1)
C(24)	24(1)	29(1)	24(1)	−1(1)	7(1)	1(1)
C(25)	27(1)	31(1)	22(1)	−5(1)	10(1)	−2(1)
C(26)	26(1)	32(1)	34(1)	−7(1)	12(1)	−3(1)
C(27)	37(1)	36(1)	39(1)	−13(1)	17(1)	−2(1)
C(28)	32(1)	28(1)	24(1)	−5(1)	5(1)	3(1)
C(29)	46(1)	43(1)	28(1)	4(1)	4(1)	6(1)
C(30)	34(1)	48(1)	35(1)	5(1)	0(1)	−15(1)
C(31)	43(1)	35(1)	28(1)	−2(1)	4(1)	−8(1)
C(32)	31(1)	28(1)	27(1)	0(1)	8(1)	−3(1)
C(33)	24(1)	23(1)	20(1)	6(1)	5(1)	1(1)
C(34)	26(1)	20(1)	19(1)	2(1)	8(1)	2(1)
C(35)	27(1)	18(1)	22(1)	−1(1)	10(1)	1(1)
C(36)	23(1)	19(1)	19(1)	2(1)	7(1)	2(1)

C(37)	25(1)	19(1)	20(1)	0(1)	9(1)	1(1)
C(38)	22(1)	29(1)	22(1)	2(1)	4(1)	−2(1)
C(39)	28(1)	30(1)	18(1)	4(1)	6(1)	0(1)
C(40)	26(1)	22(1)	21(1)	0(1)	9(1)	−1(1)
C(41)	29(1)	26(1)	20(1)	−1(1)	10(1)	−4(1)
C(42)	35(1)	30(1)	29(1)	4(1)	14(1)	−1(1)
C(43)	52(1)	32(1)	33(1)	7(1)	20(1)	−3(1)
C(44)	48(1)	44(1)	34(1)	−3(1)	24(1)	−16(1)
C(45)	38(1)	53(1)	50(1)	−6(1)	22(1)	5(1)
C(46)	46(1)	32(1)	28(1)	−4(1)	21(1)	2(1)
C(47)	29(1)	26(1)	22(1)	0(1)	10(1)	−4(1)
C(48)	27(1)	21(1)	19(1)	0(1)	8(1)	−3(1)
C(49)	28(1)	18(1)	20(1)	0(1)	10(1)	2(1)
C(50)	24(1)	56(1)	35(1)	8(1)	5(1)	−5(1)
C(51)	26(1)	37(1)	27(1)	4(1)	6(1)	2(1)
C(52)	25(1)	28(1)	24(1)	−3(1)	8(1)	5(1)
C(53)	25(1)	23(1)	21(1)	3(1)	8(1)	−4(1)
C(54)	31(1)	35(1)	27(1)	9(1)	8(1)	3(1)
C(55)	32(1)	63(1)	47(1)	8(1)	18(1)	−6(1)
C(56)	31(1)	49(1)	39(1)	14(1)	12(1)	1(1)
C(57)	26(1)	29(1)	20(1)	−2(1)	5(1)	−7(1)
C(58)	30(1)	26(1)	17(1)	1(1)	6(1)	−4(1)

Table S5. Hydrogen coordinates (×10[4]) and isotropic displacement parameters (Å^2 × 10^3) for cyc43.

	x	*y*	*z*	U(eq)
H(1A)	−1838	3154	8042	104
H(1B)	−1842	2079	7486	104
H(1C)	−2462	2547	7557	104
H(2A)	−2332	3586	6601	56
H(2B)	−2330	4666	7159	56
H(5)	−283	3960	7278	29
H(8)	1268	4235	7990	33
H(9)	1875	2690	7704	32
H(12)	2627	2166	7254	35
H(13)	3235	630	6956	41
H(14)	2817	−720	5993	44
H(15A)	−560	5680	8149	31
H(15B)	−125	4430	8396	31
H(18)	1197	8005	8922	39
H(19)	1495	9034	9989	50
H(20)	1057	8412	10,801	52
H(21)	324	6747	10,535	48
H(22)	13	5728	9460	38
H(23)	943	6888	7942	29
H(24)	255	4684	5981	31
H(25)	873	3165	5694	32
H(26)	1171	972	5639	36
H(27)	1783	−543	5333	44
H(29A)	−1374	7308	5786	61
H(29B)	−840	7194	5486	61
H(29C)	−823	8314	6025	61
H(30)	2165	3526	5381	50

H(31)	3058	3341	5122	45
H(32)	3908	4611	5714	35
H(35)	4747	5645	6449	26
H(38)	4854	8030	5922	30
H(39)	5419	9296	5424	31
H(42)	5850	11,504	5368	37
H(43)	6422	12,756	4867	45
H(44)	7461	12,364	5153	48
H(45A)	3333	9930	8630	68
H(45B)	3710	11,113	8477	68
H(45C)	3564	11,096	9167	68
H(46A)	4573	10,429	9411	40
H(46B)	4192	9213	9533	40
H(49)	4732	8947	7500	26
H(50)	2111	5066	6192	47
H(51)	2941	6418	6749	37
H(52A)	3886	7119	7578	31
H(52B)	3769	8205	6990	31
H(54A)	5781	6221	9406	47
H(54B)	6297	6280	9075	47
H(54C)	5847	5057	8926	47
H(55)	7925	10,704	5935	55
H(56)	7359	9443	6438	48
H(57)	6792	9491	7256	31
H(58)	6219	8265	7760	30

II. Copies of ^{1}H- and ^{13}C-NMR Spectra

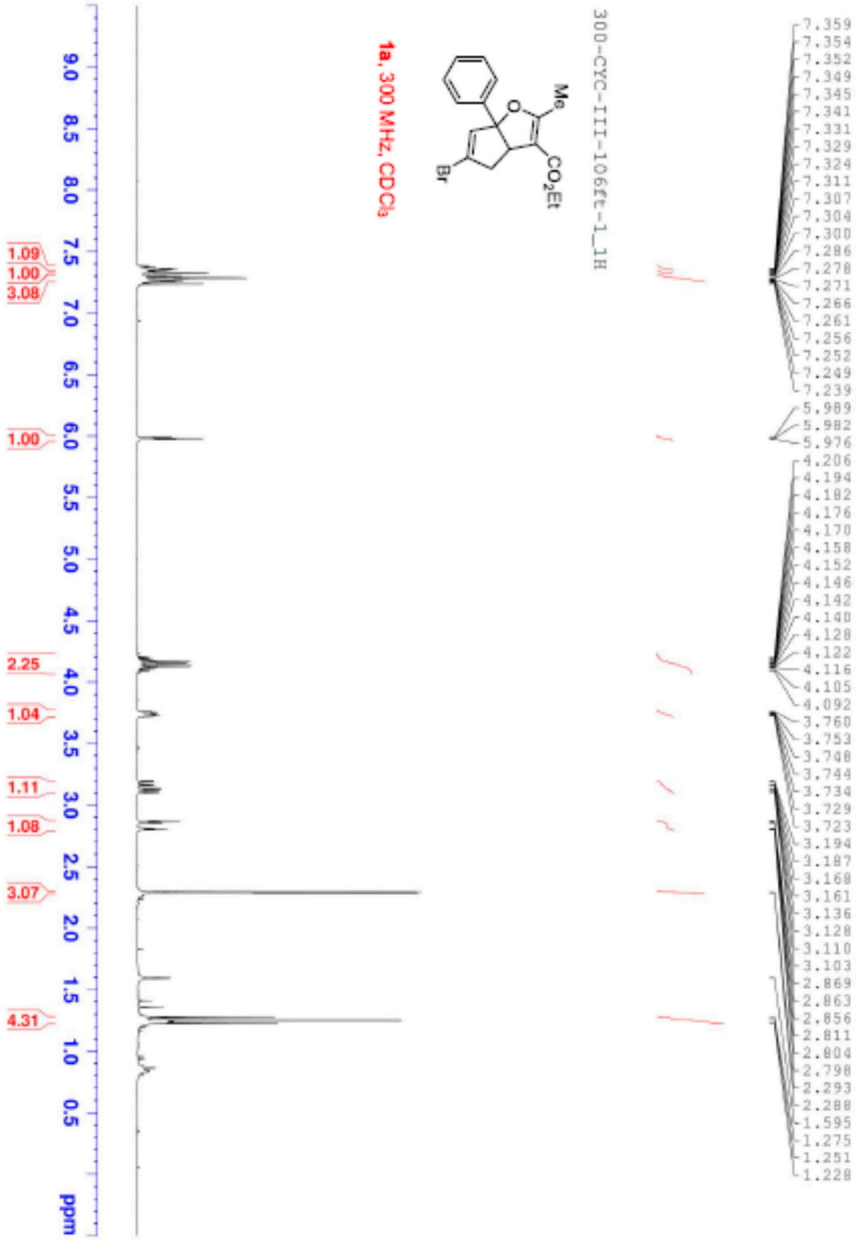

Figure S2. ^{1}H-NMR of **1a**.

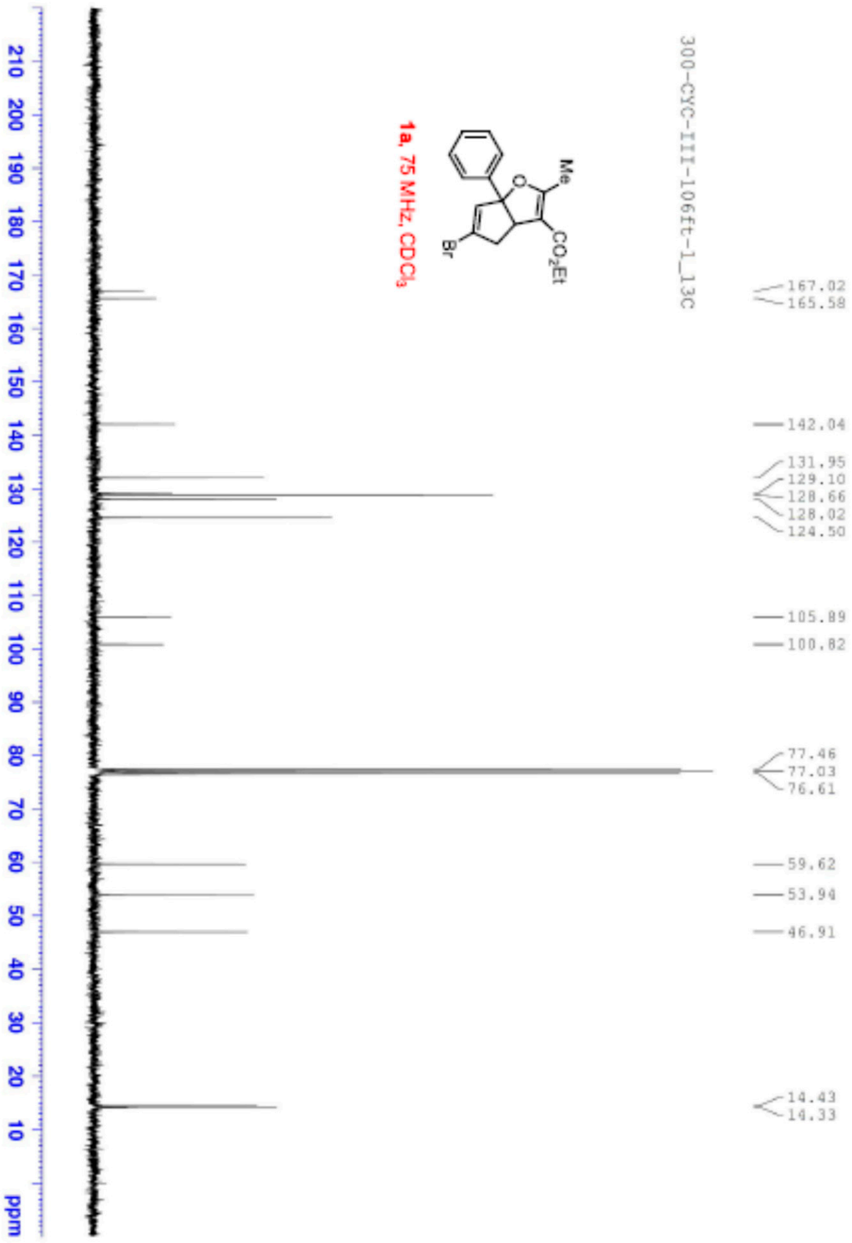

Figure S3. ^{13}C-NMR of **1a**.

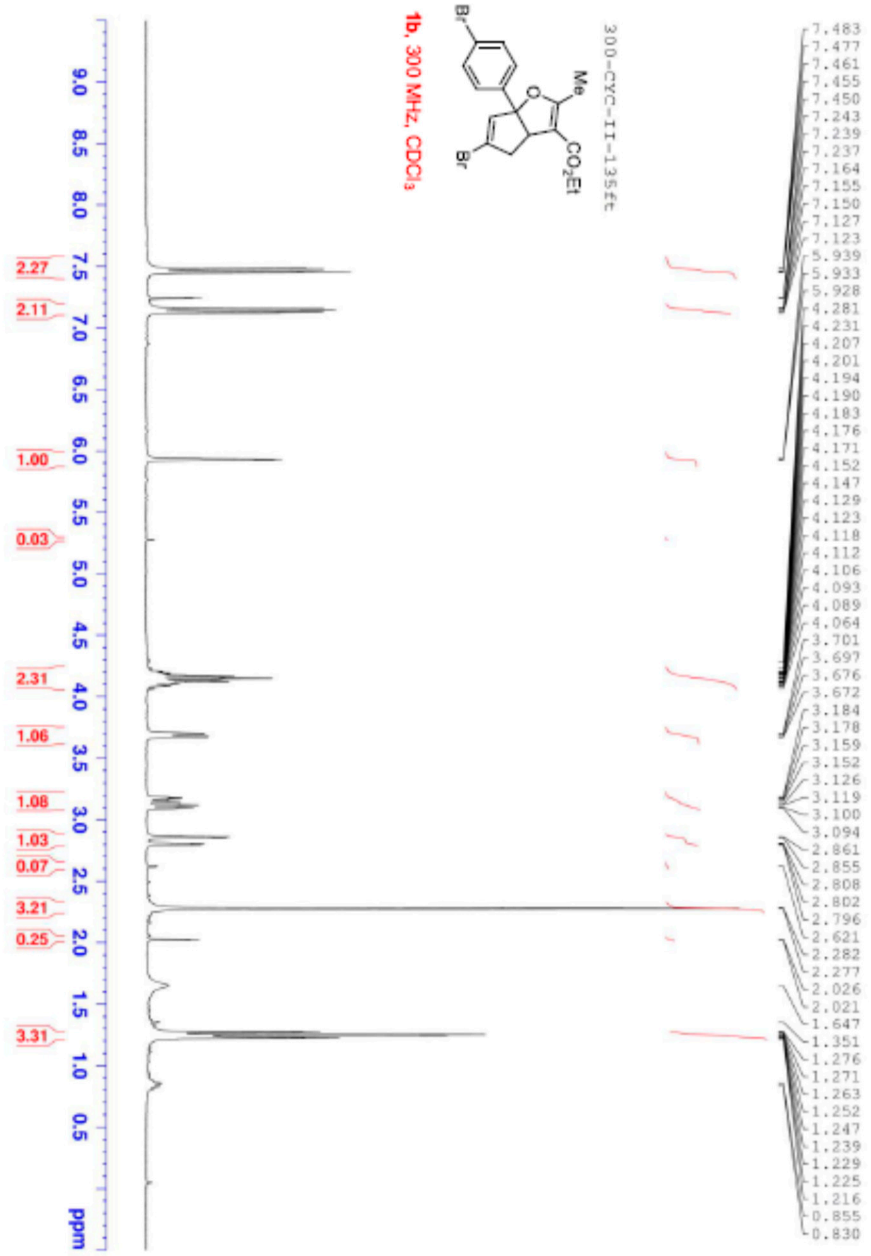

Figure S4. ^{1}H-NMR of **1b**.

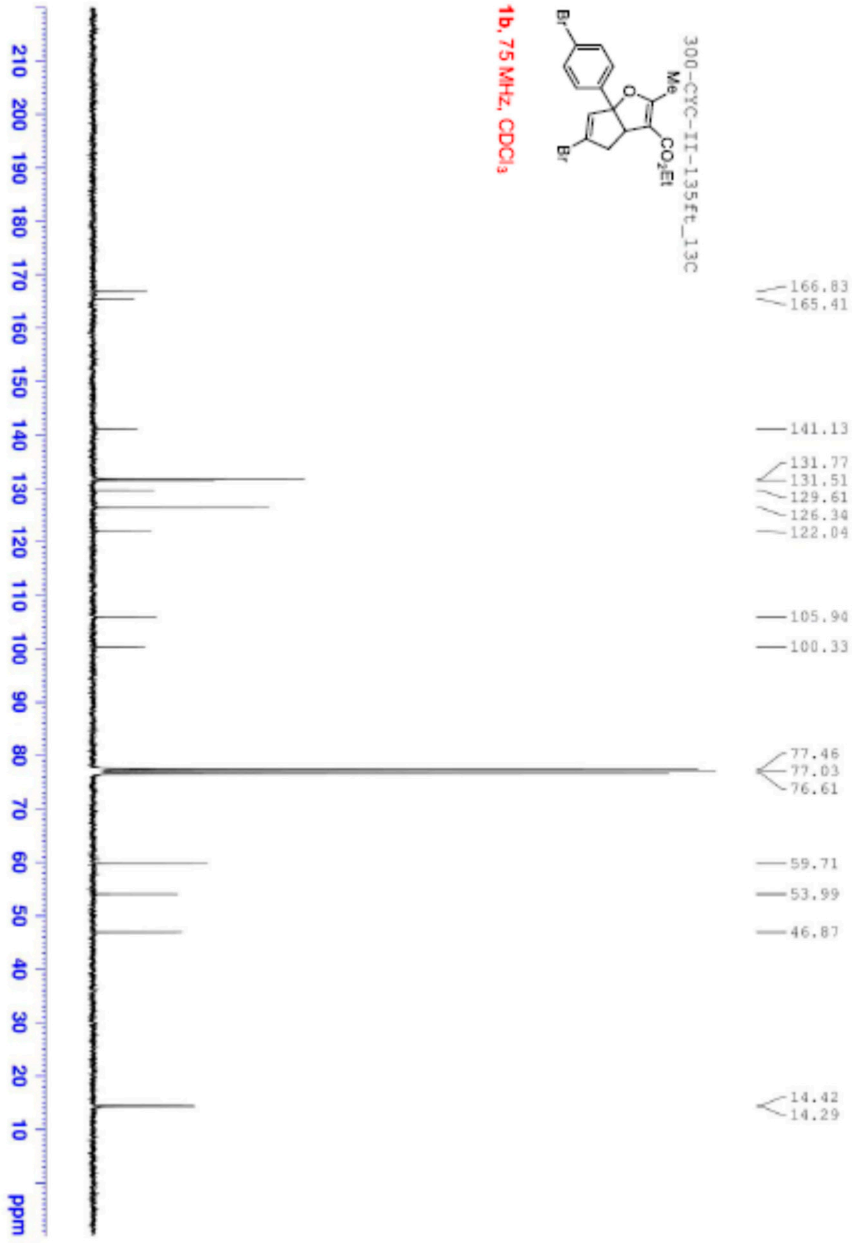

Figure S5. ^{13}C-NMR of **1b**.

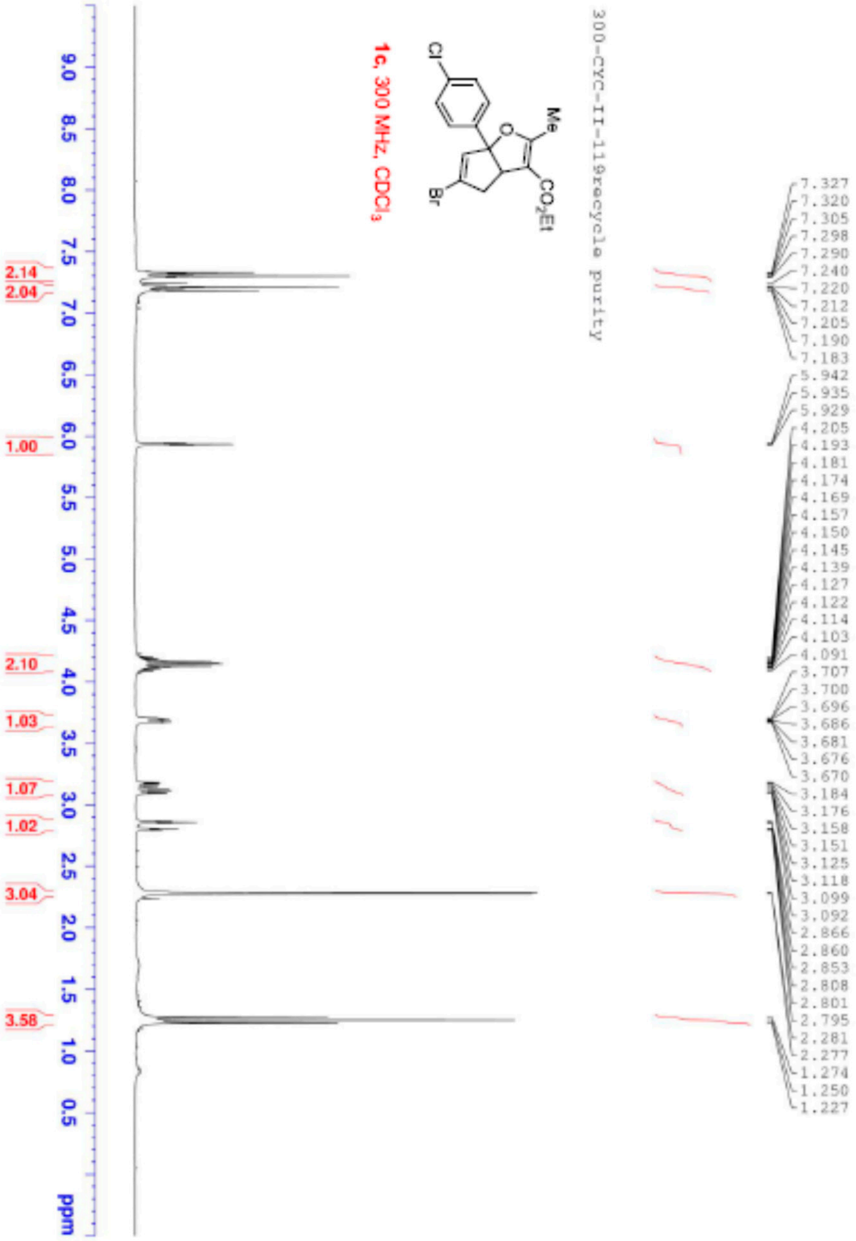

Figure S6. ^{1}H-NMR of **1c**.

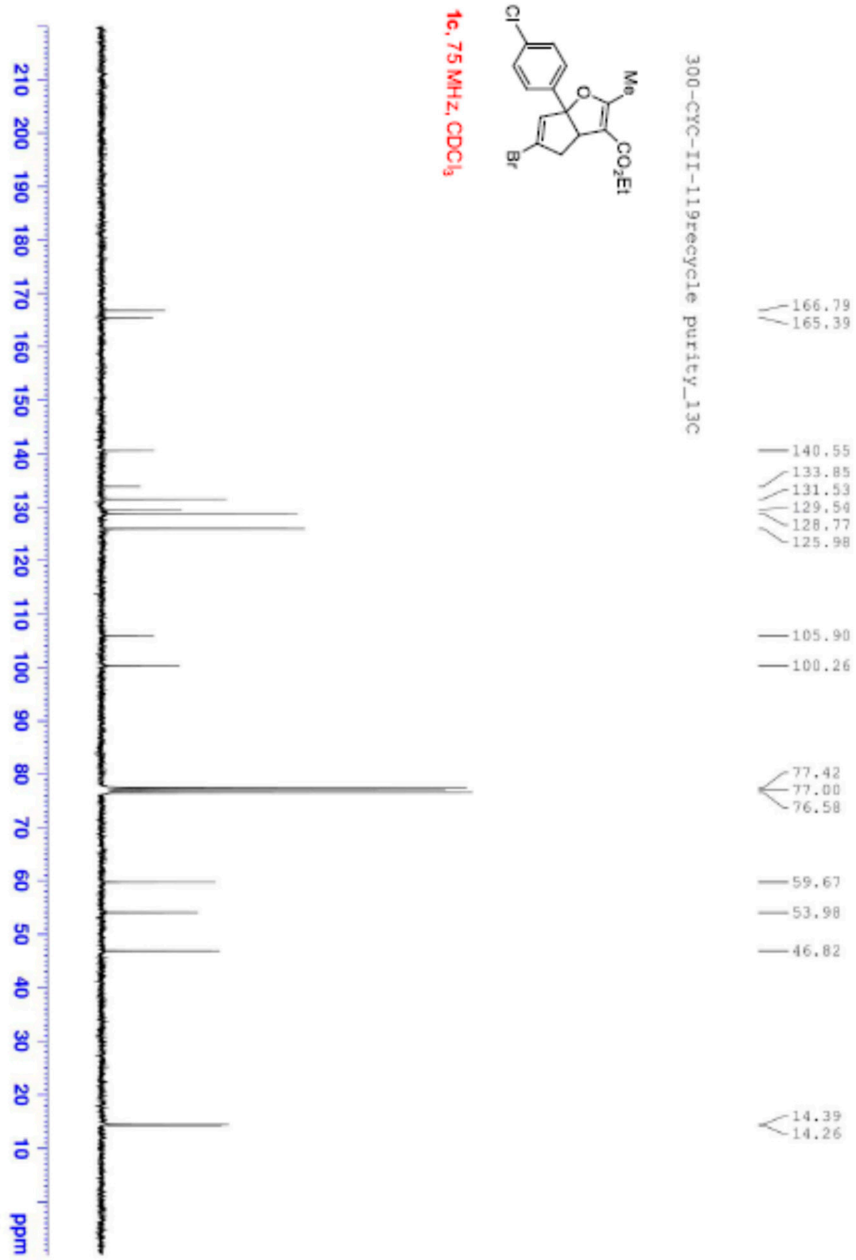

Figure S7. ^{13}C-NMR of **1c**.

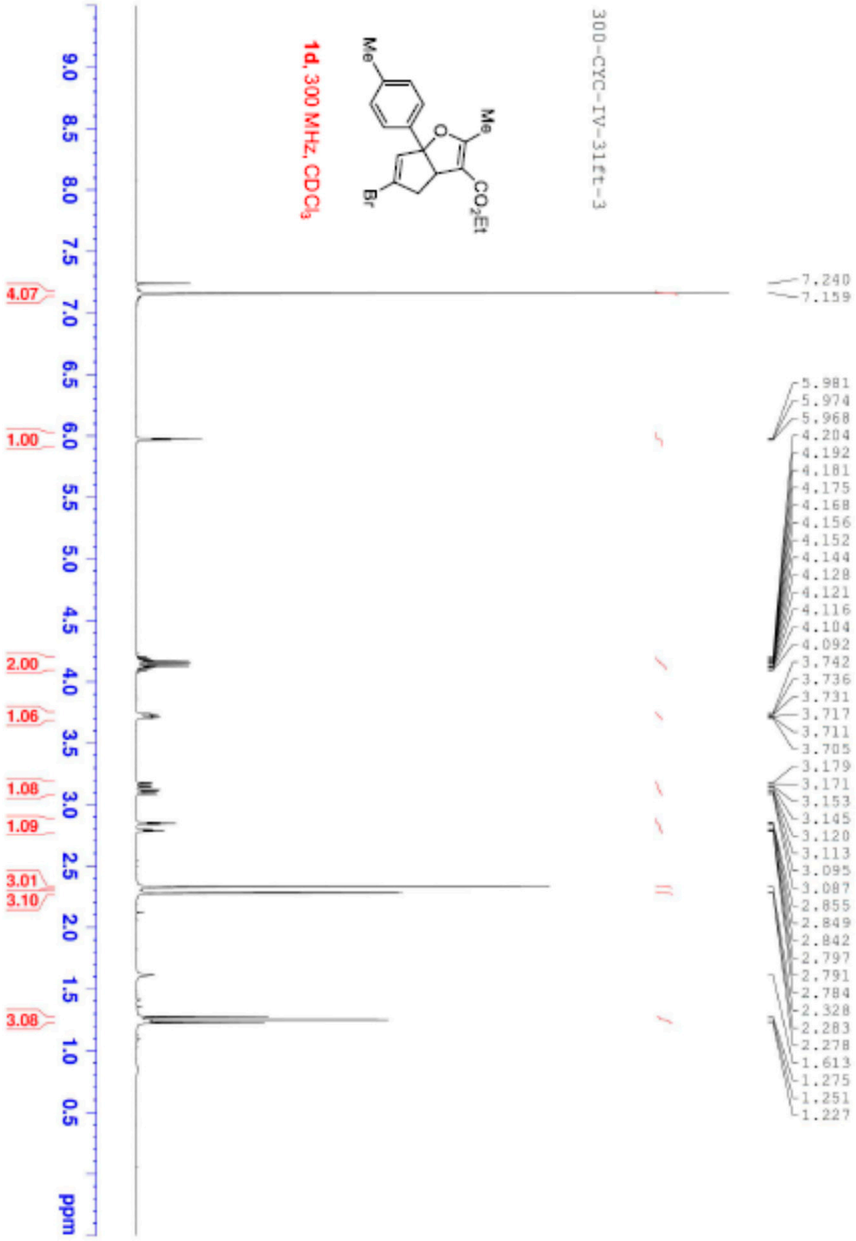

Figure S8. ^{1}H-NMR of **1d**.

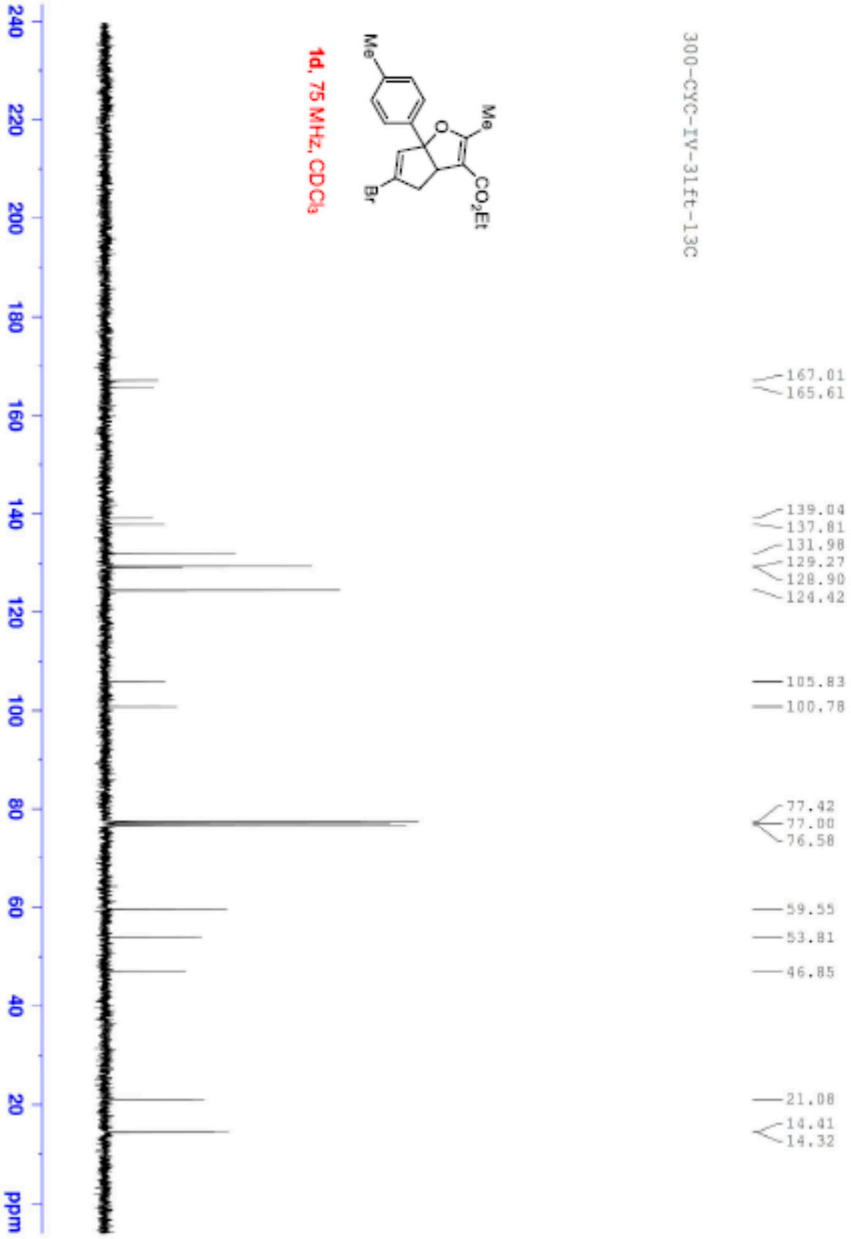

Figure S9. ^{13}C-NMR of **1d**.

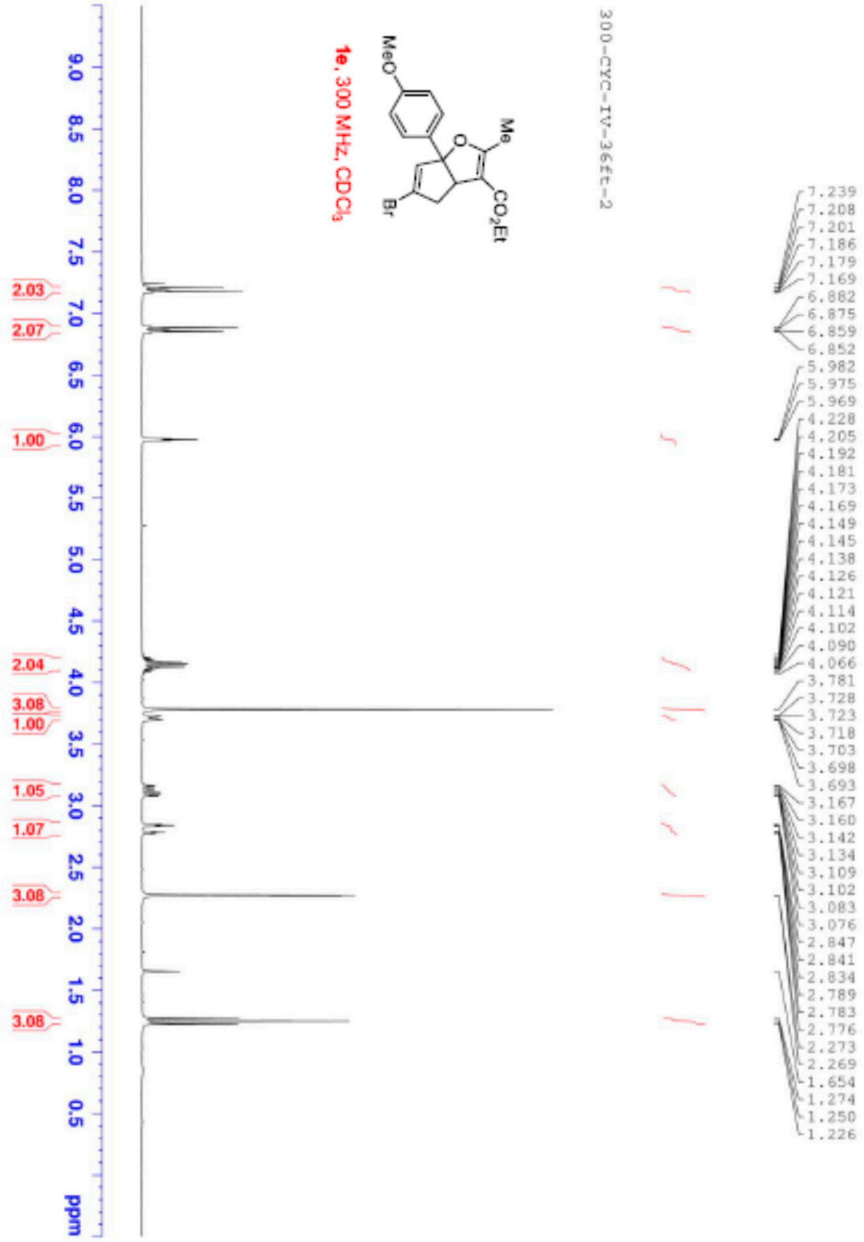

Figure S10. ^{1}H-NMR of **1e**.

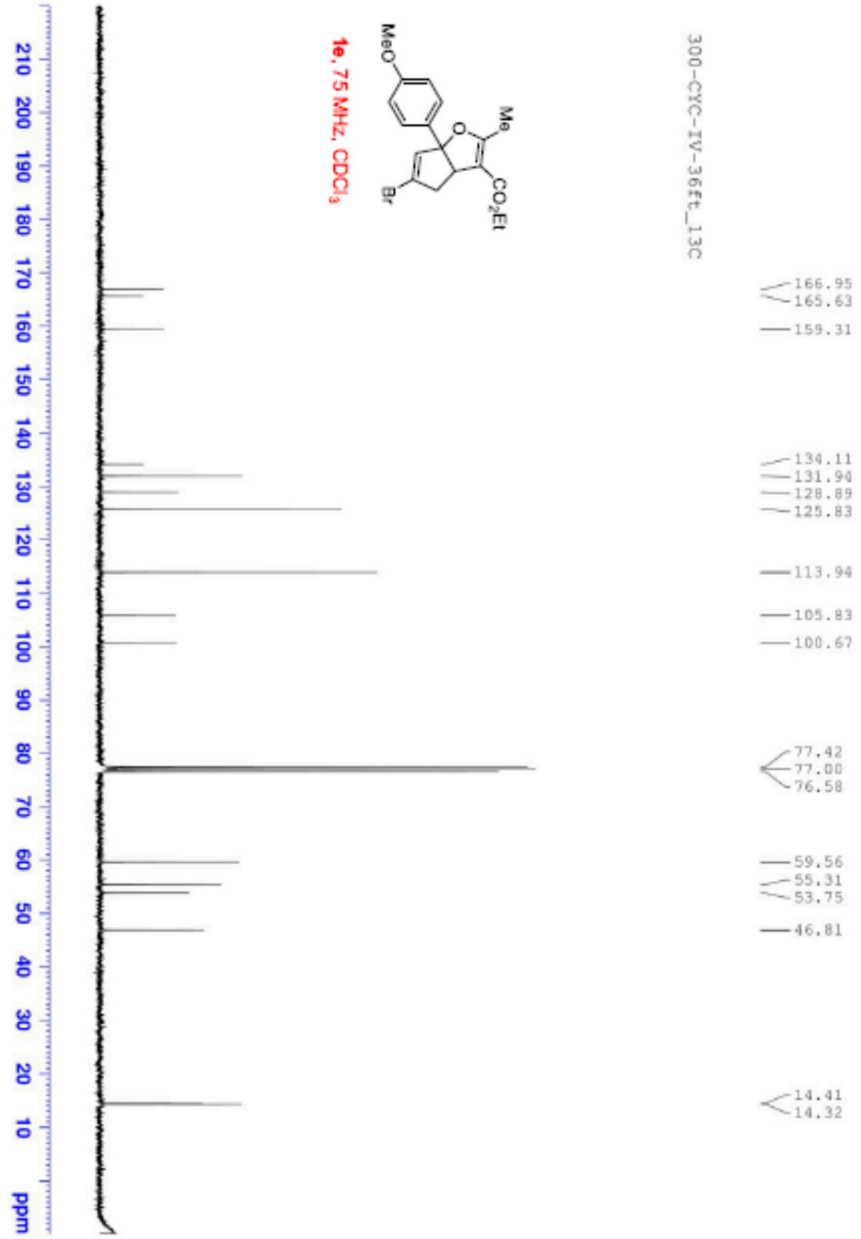

Figure S11. ^{13}C-NMR of **1e**.

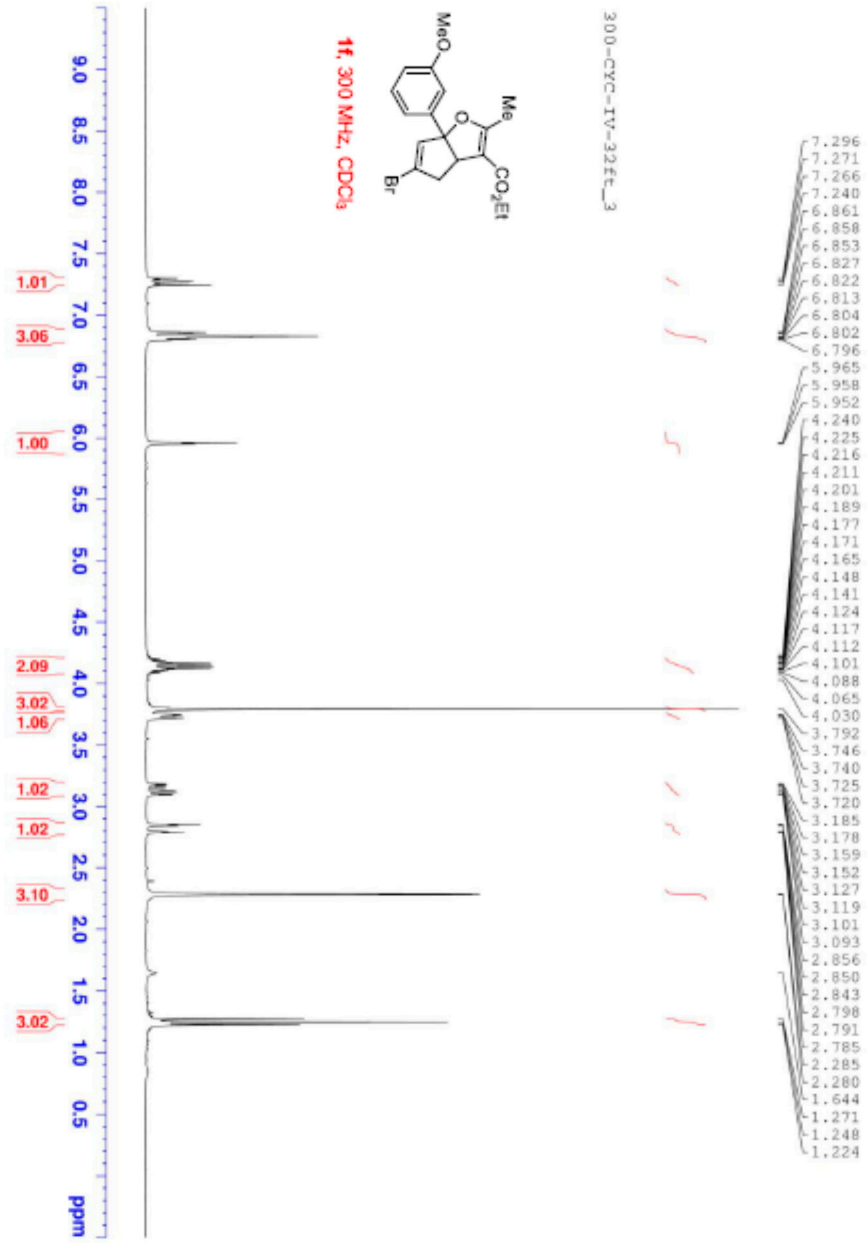

Figure S12. ^{1}H-NMR of **1f**.

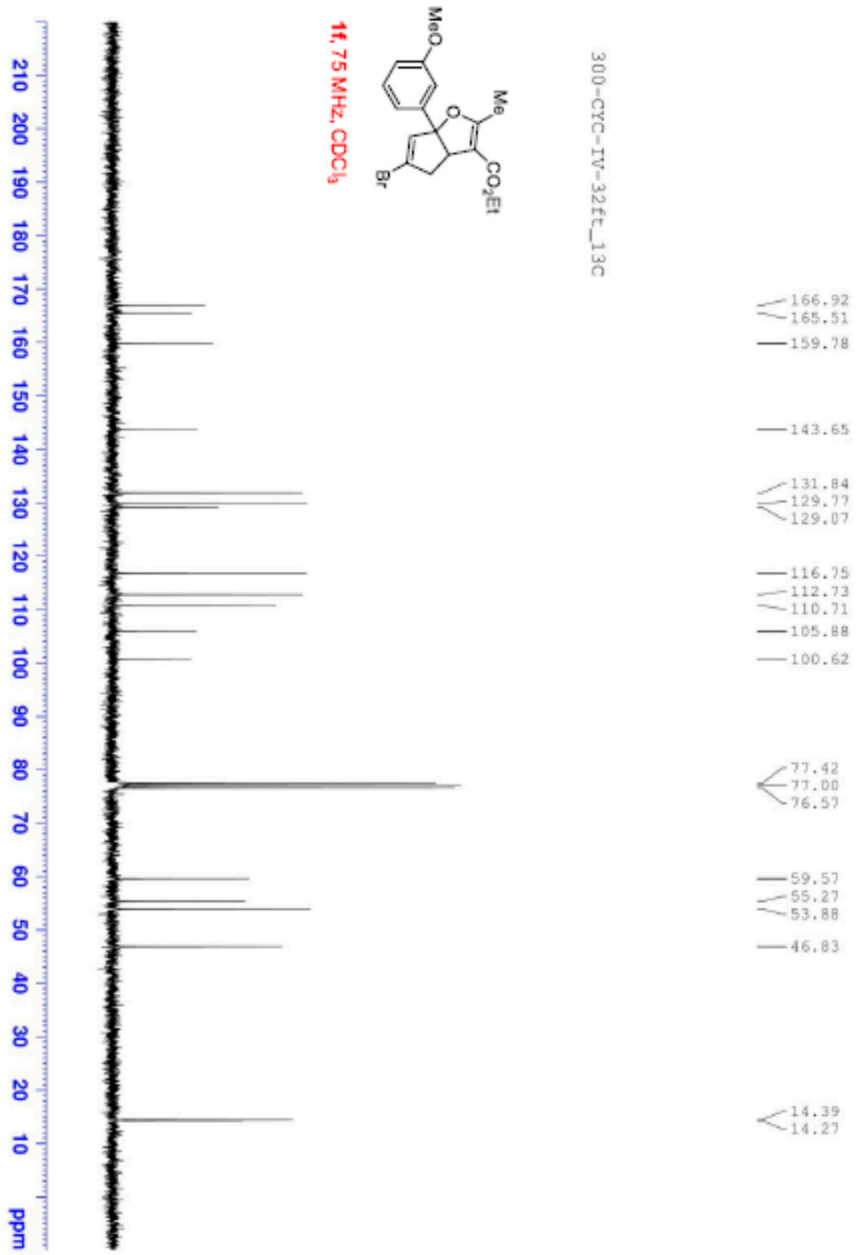

Figure S13. ^{13}C-NMR of **1f**.

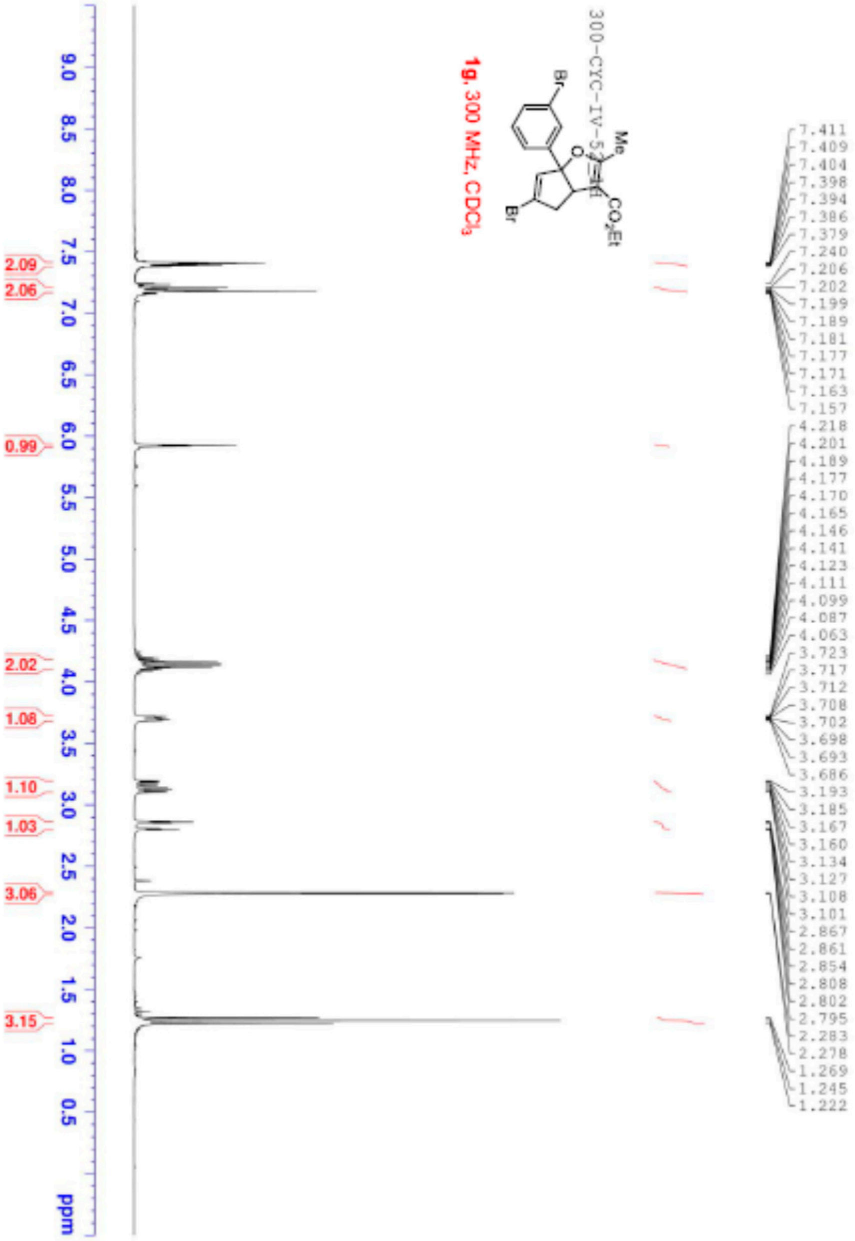

Figure S14. ^{1}H-NMR of **1g**.

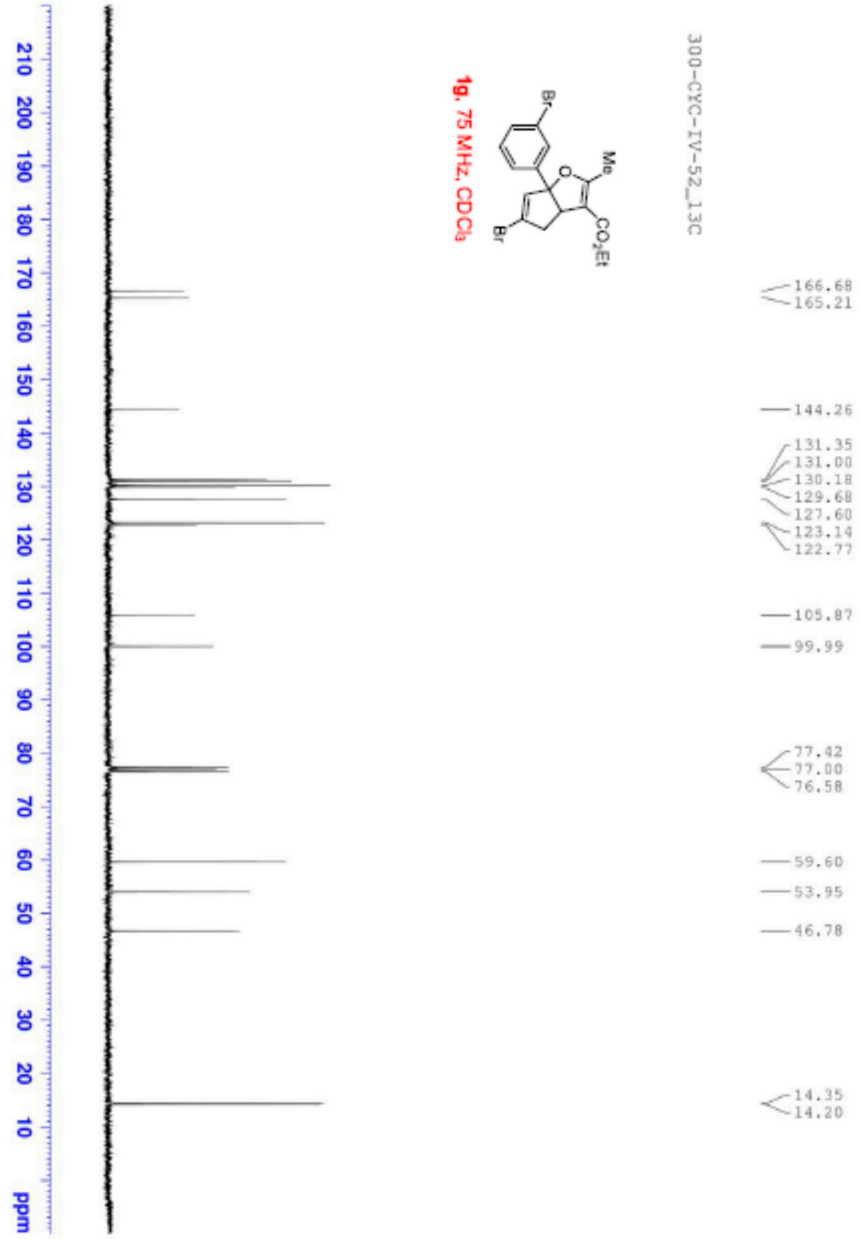

Figure S15. ^{13}C-NMR of **1g**.

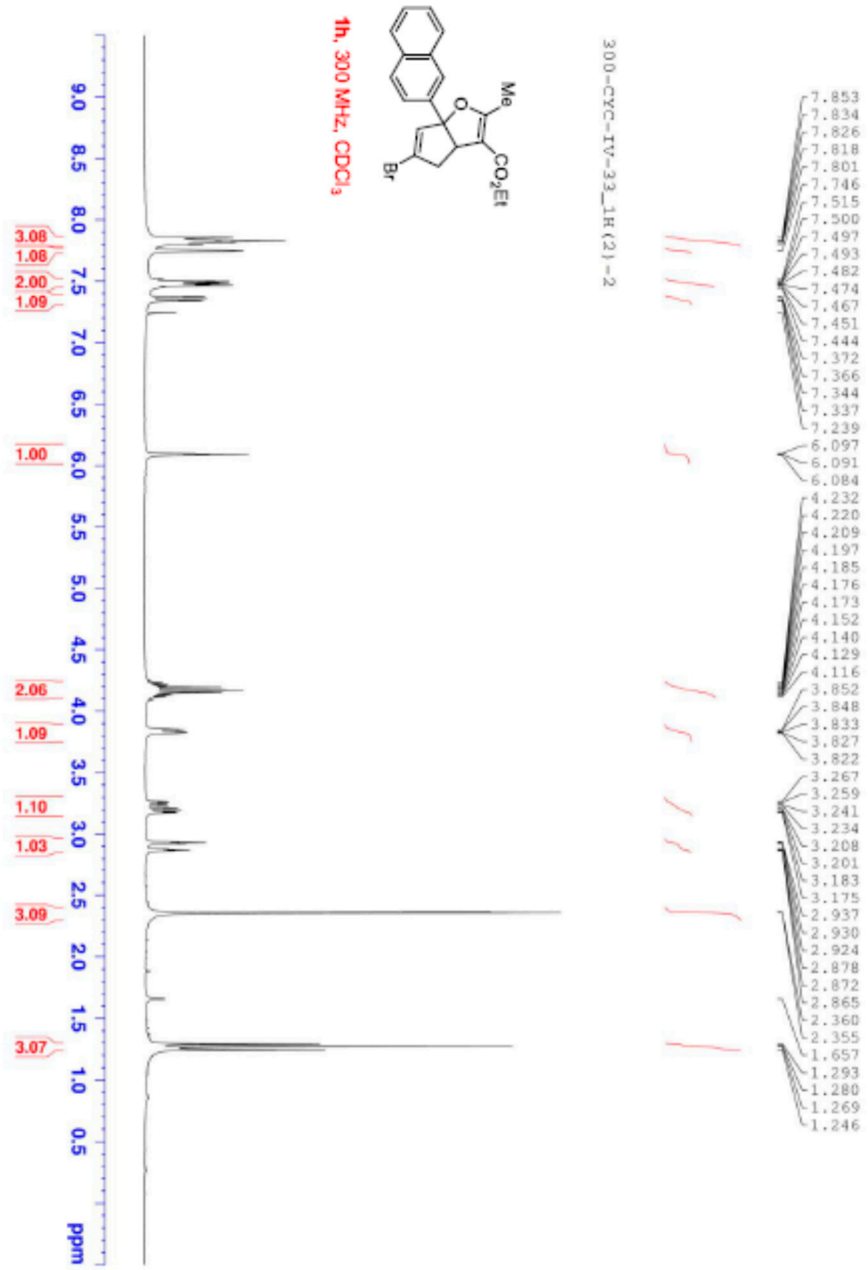

Figure S16. ^{1}H-NMR of **1h**.

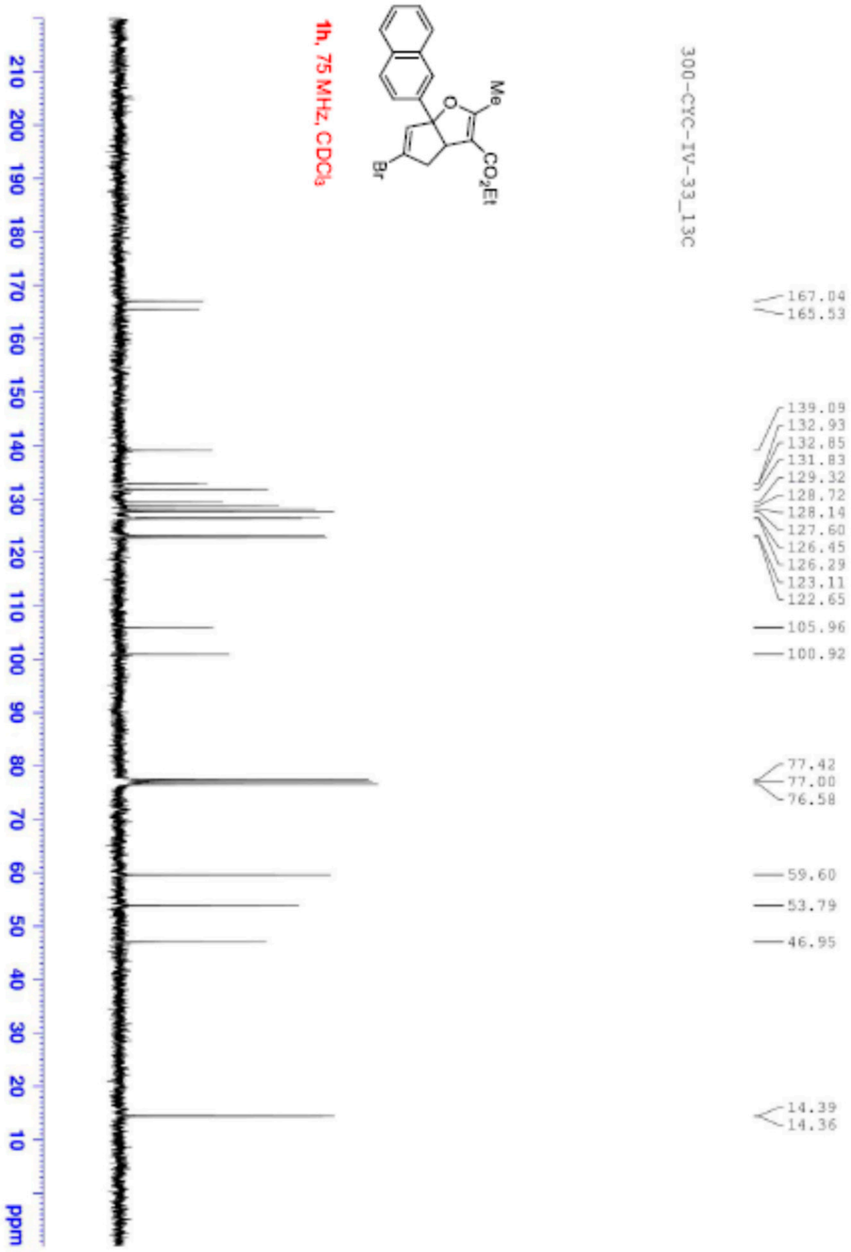

Figure S17. ^{13}C-NMR of **1h**.

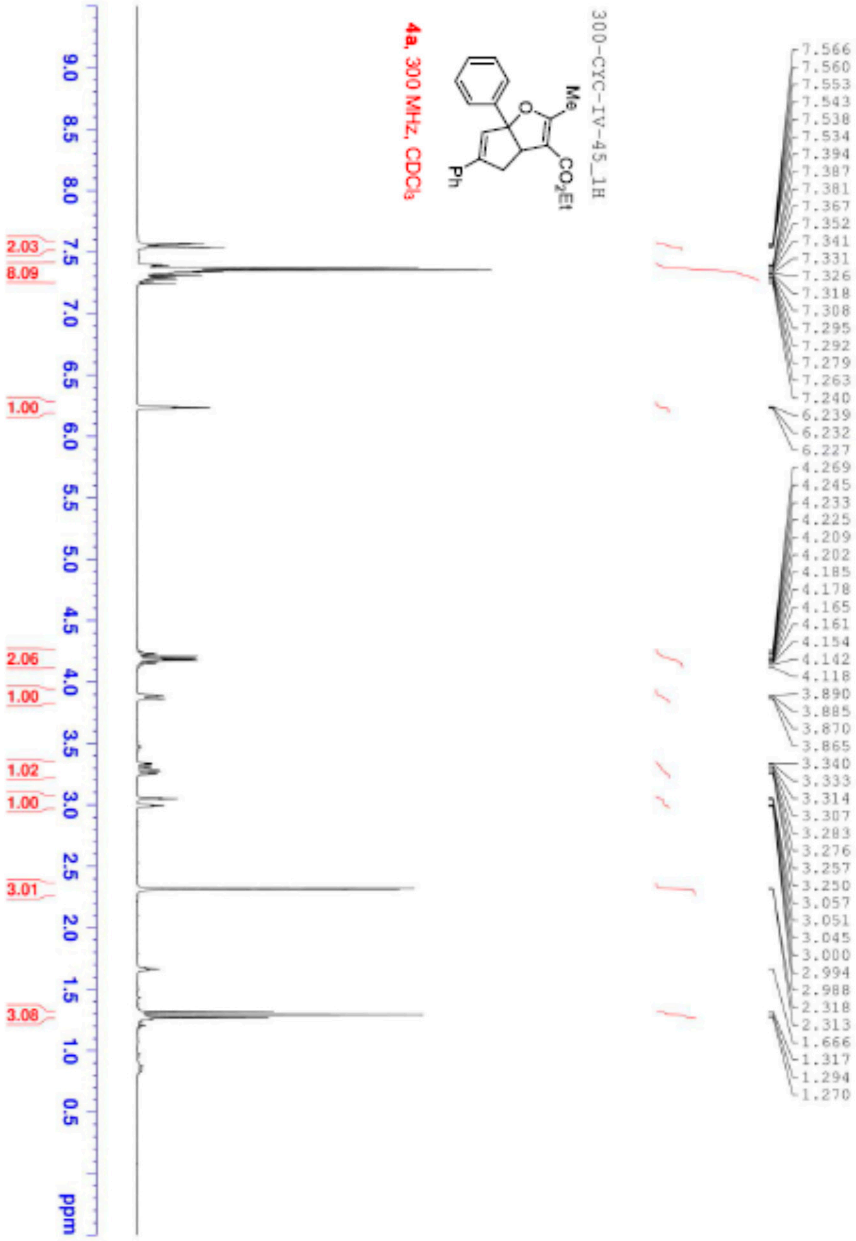

Figure S18. ^{1}H-NMR of **4a**.

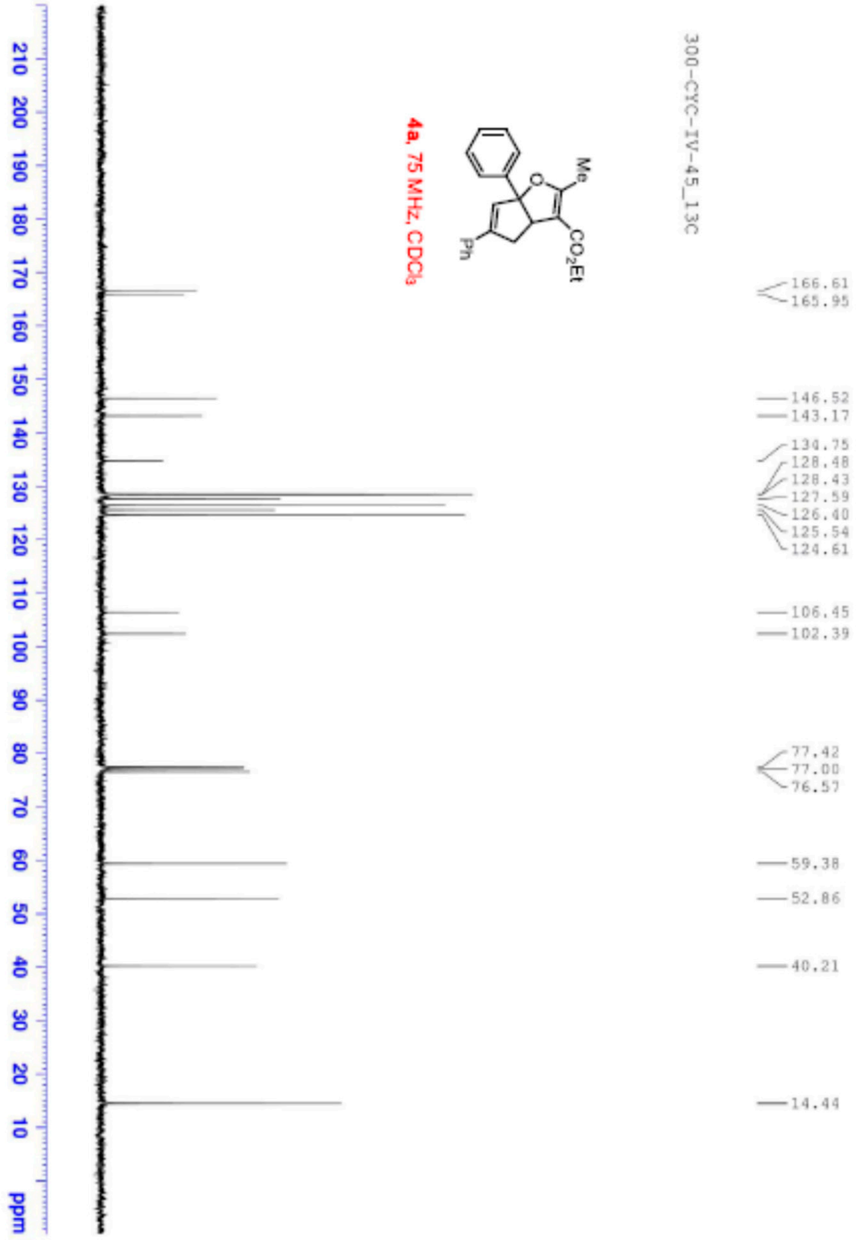

Figure S19. ^{13}C-NMR of **4a**.

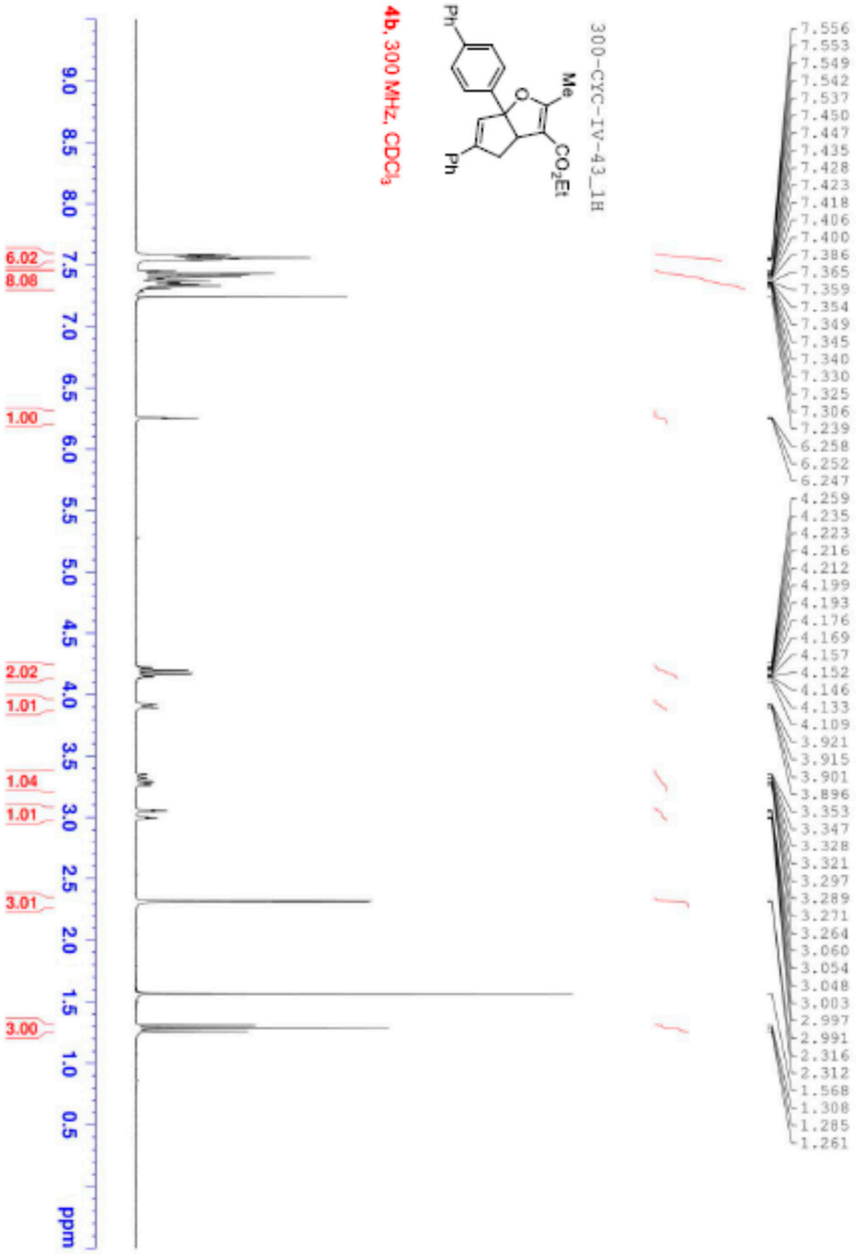

Figure S20. ^{1}H-NMR of **4b**.

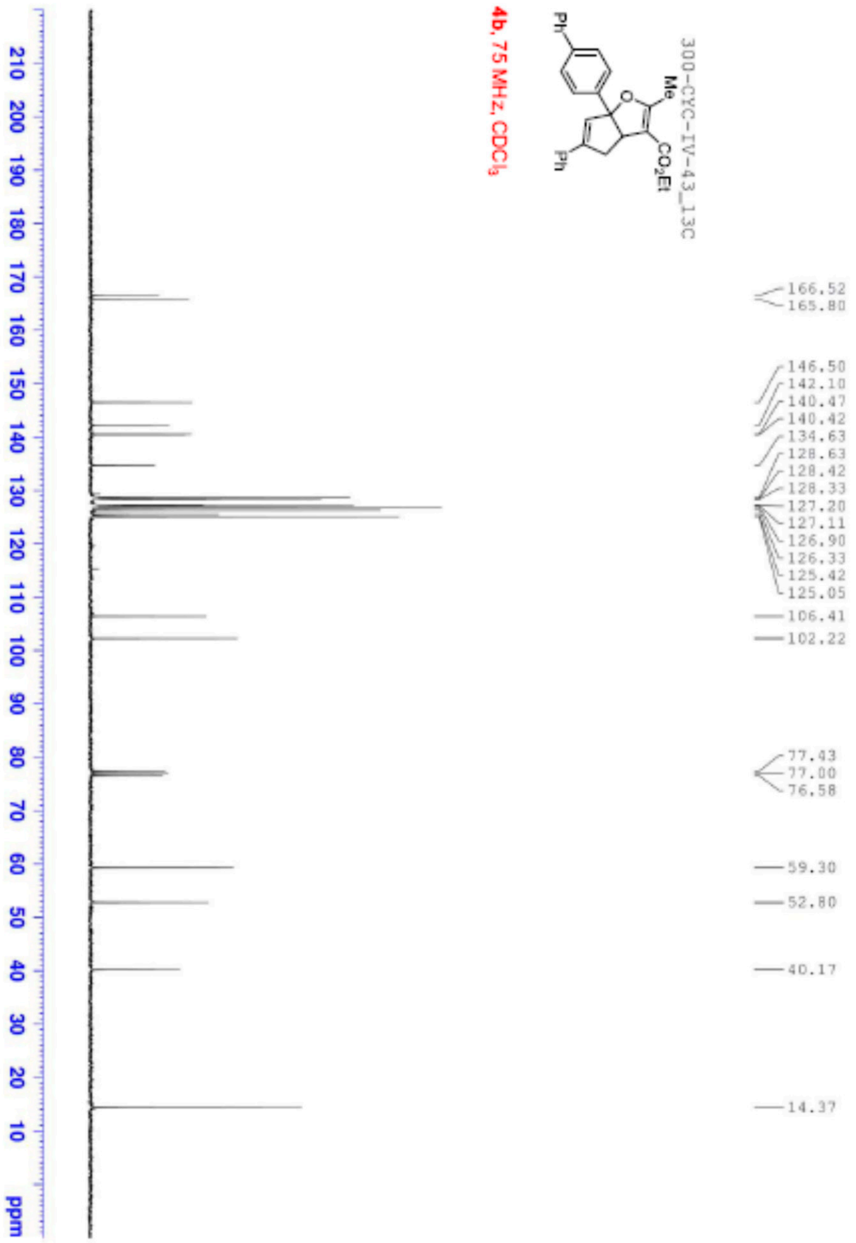

Figure S21. ^{13}C-NMR of **4b**.

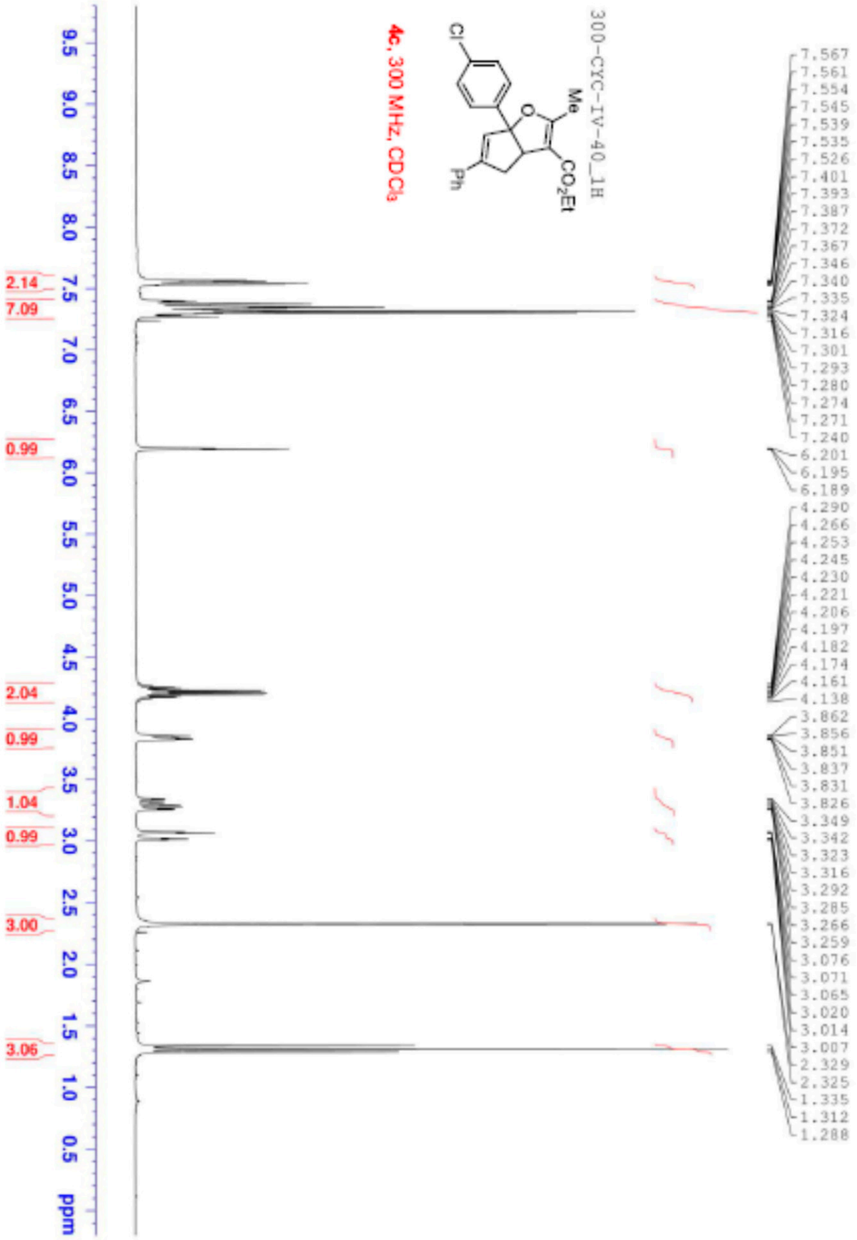

Figure S22. ¹H-NMR of **4c**.

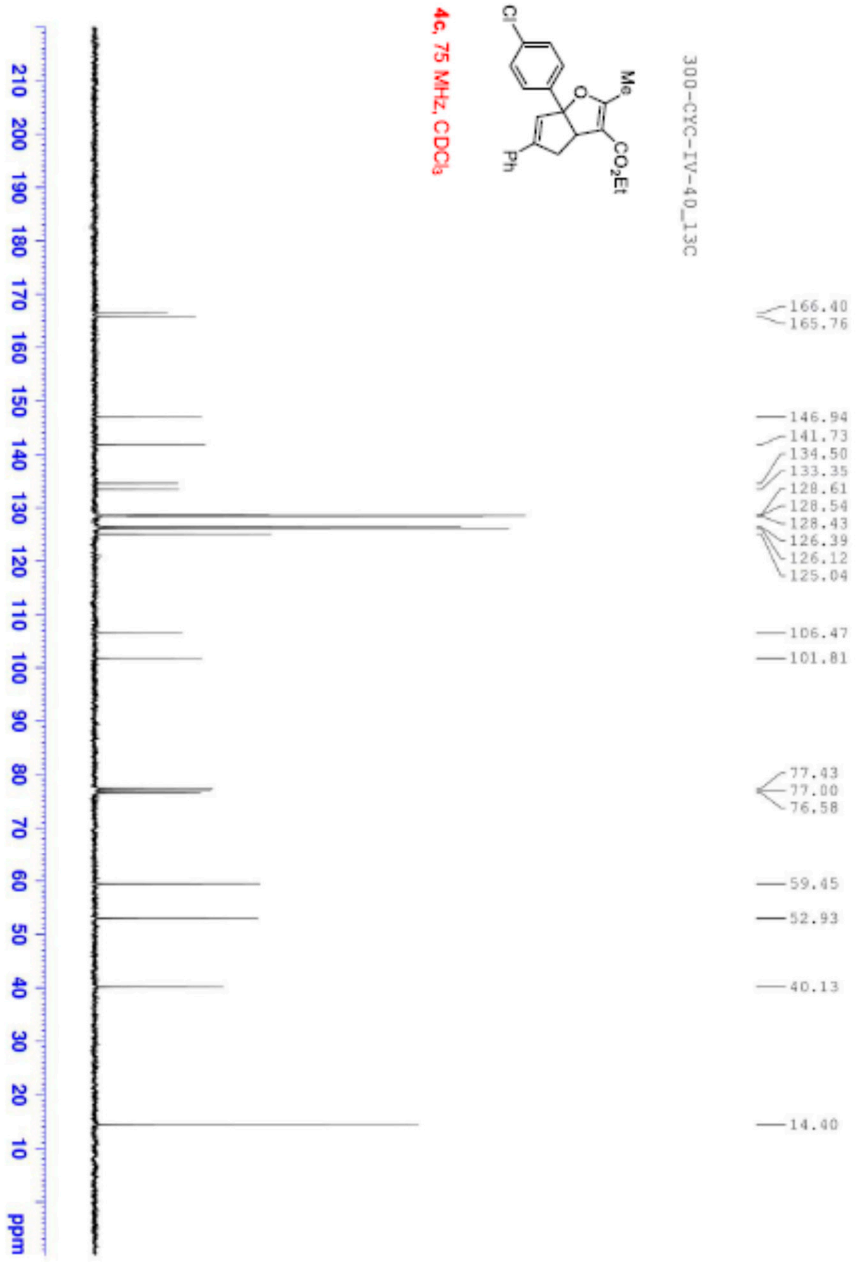

Figure S23. ^{13}C-NMR of **4c**.

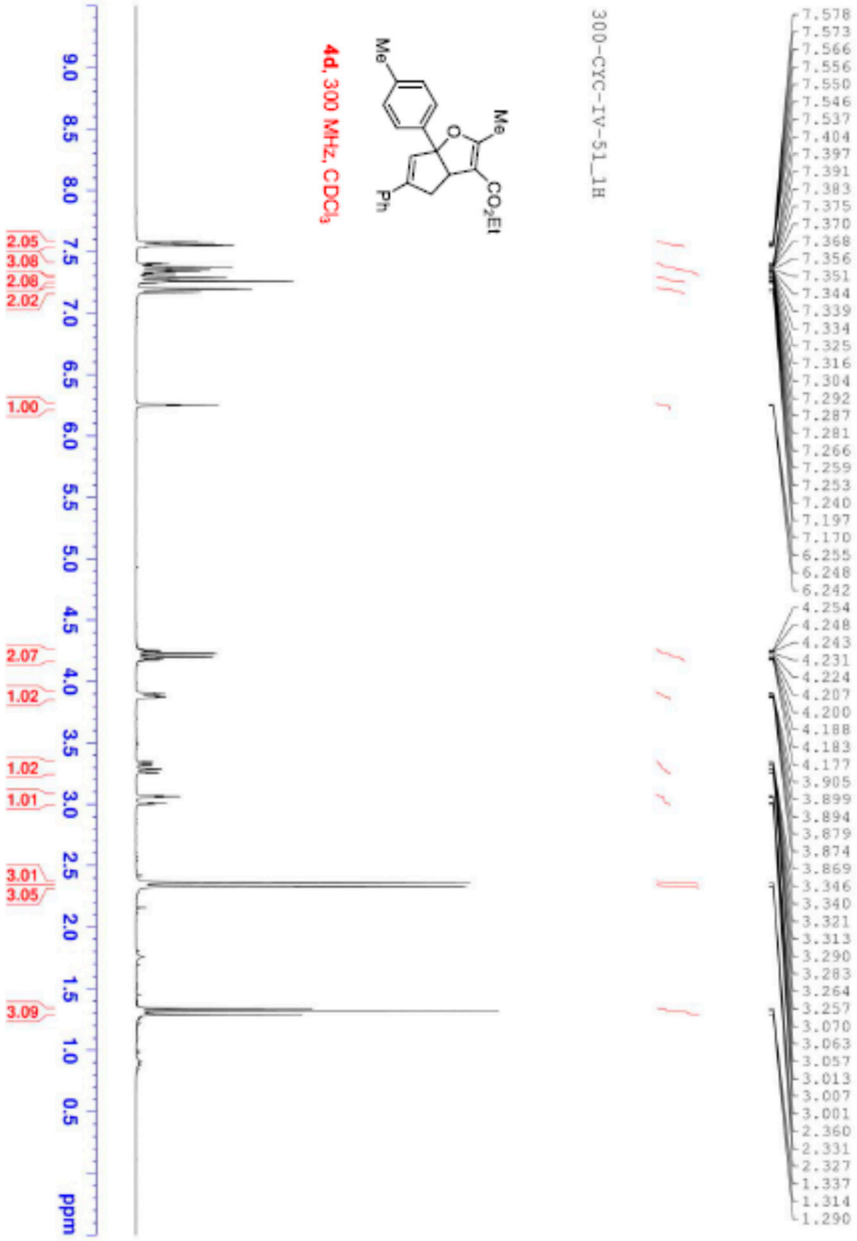

Figure S24. ^{1}H-NMR of **4d**.

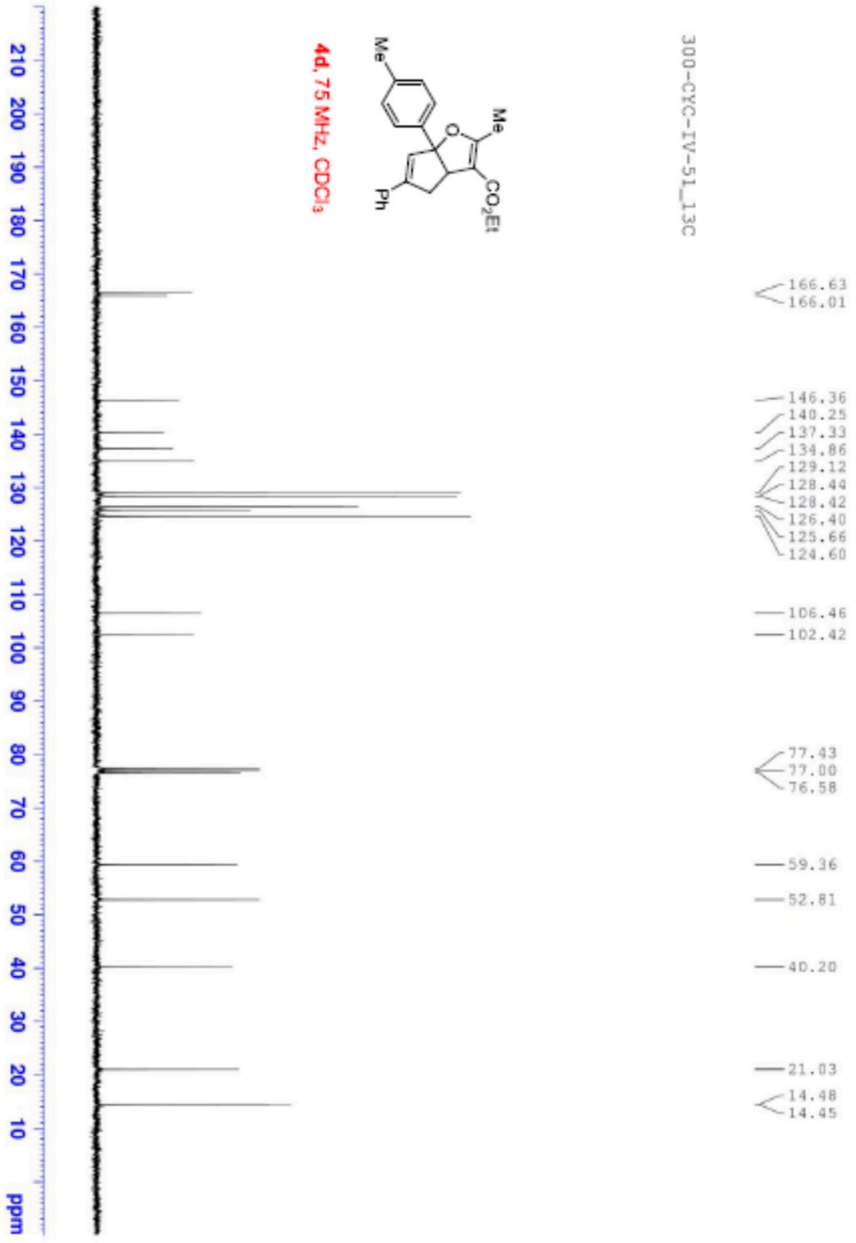

Figure S25. ^{13}C-NMR of **4d**.

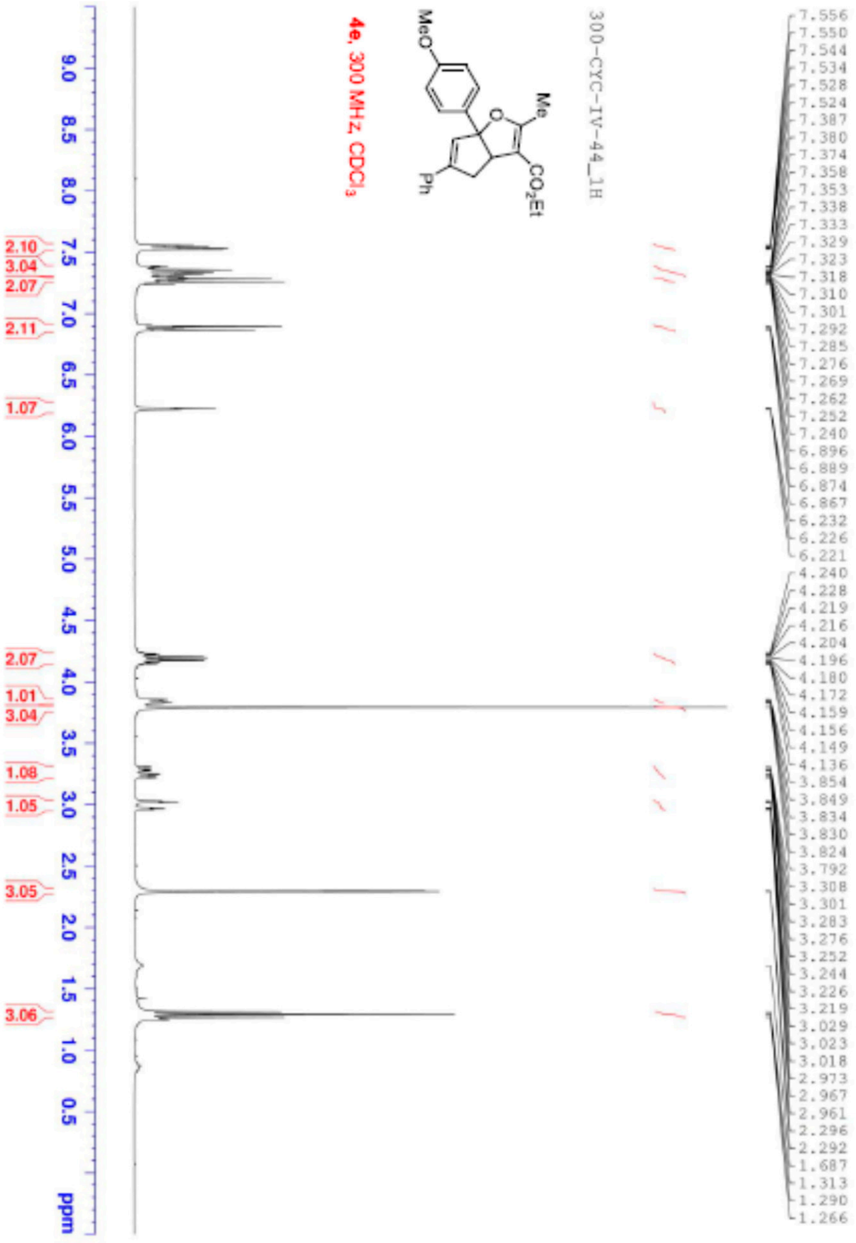

Figure S26. ^{1}H-NMR of **4e**.

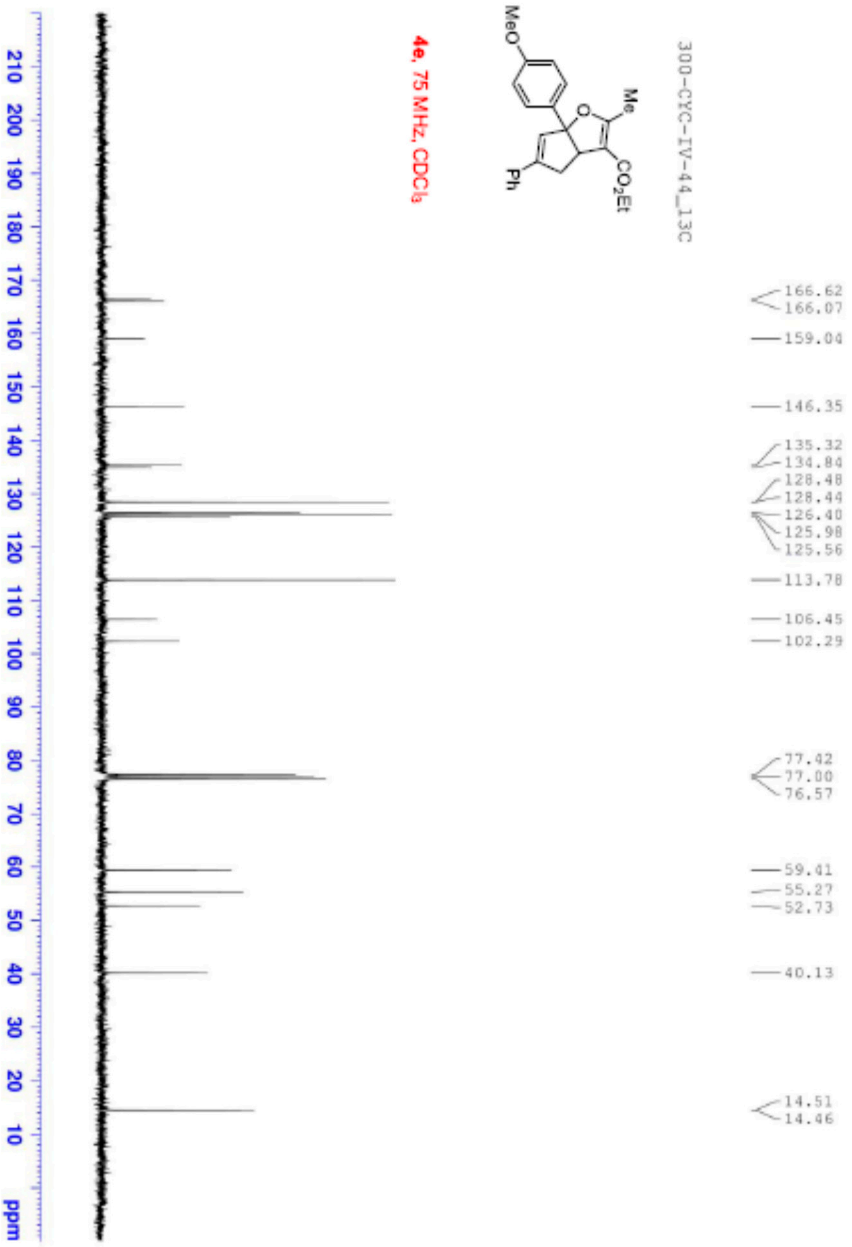

Figure S27. ^{13}C-NMR of **4e**.

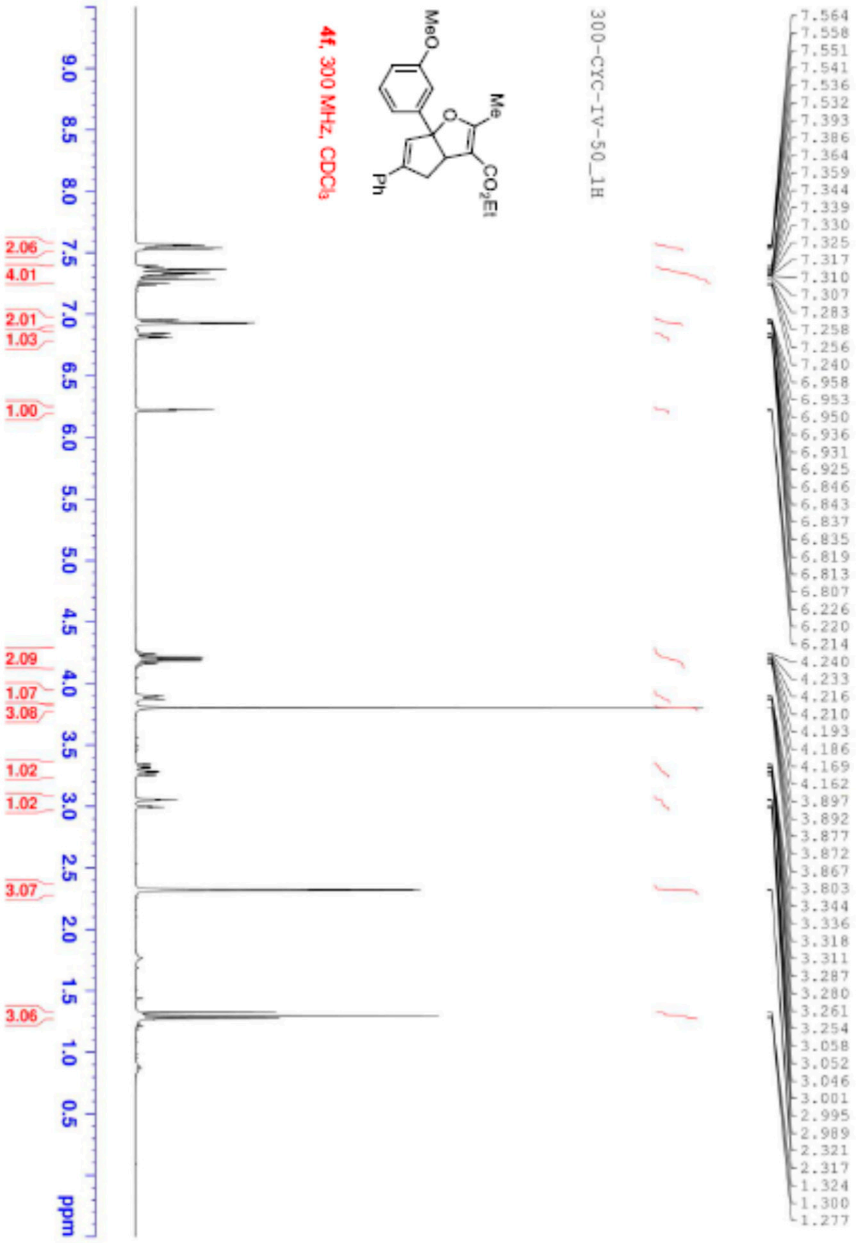

Figure S28. ^{1}H-NMR of **4f**.

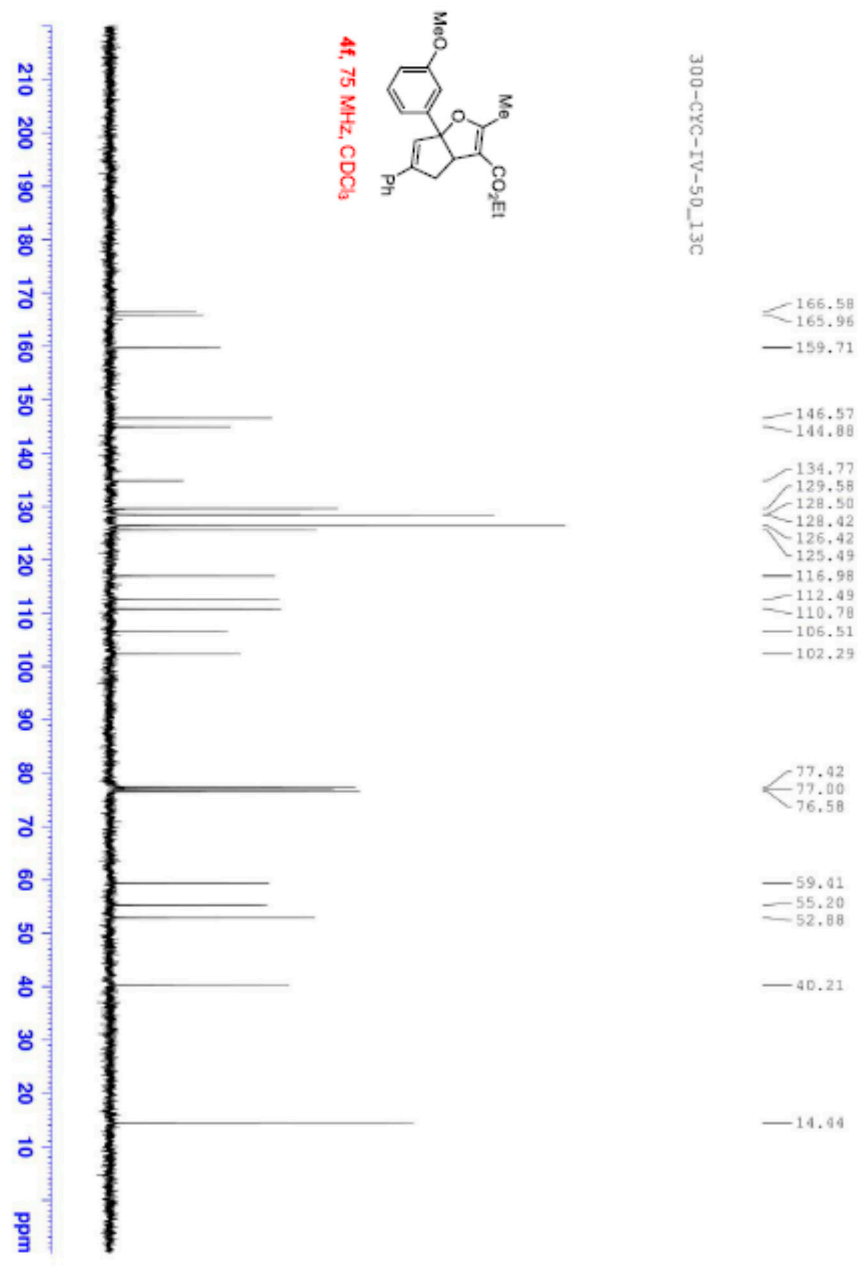

Figure S29. ^{13}C-NMR of **4f**.

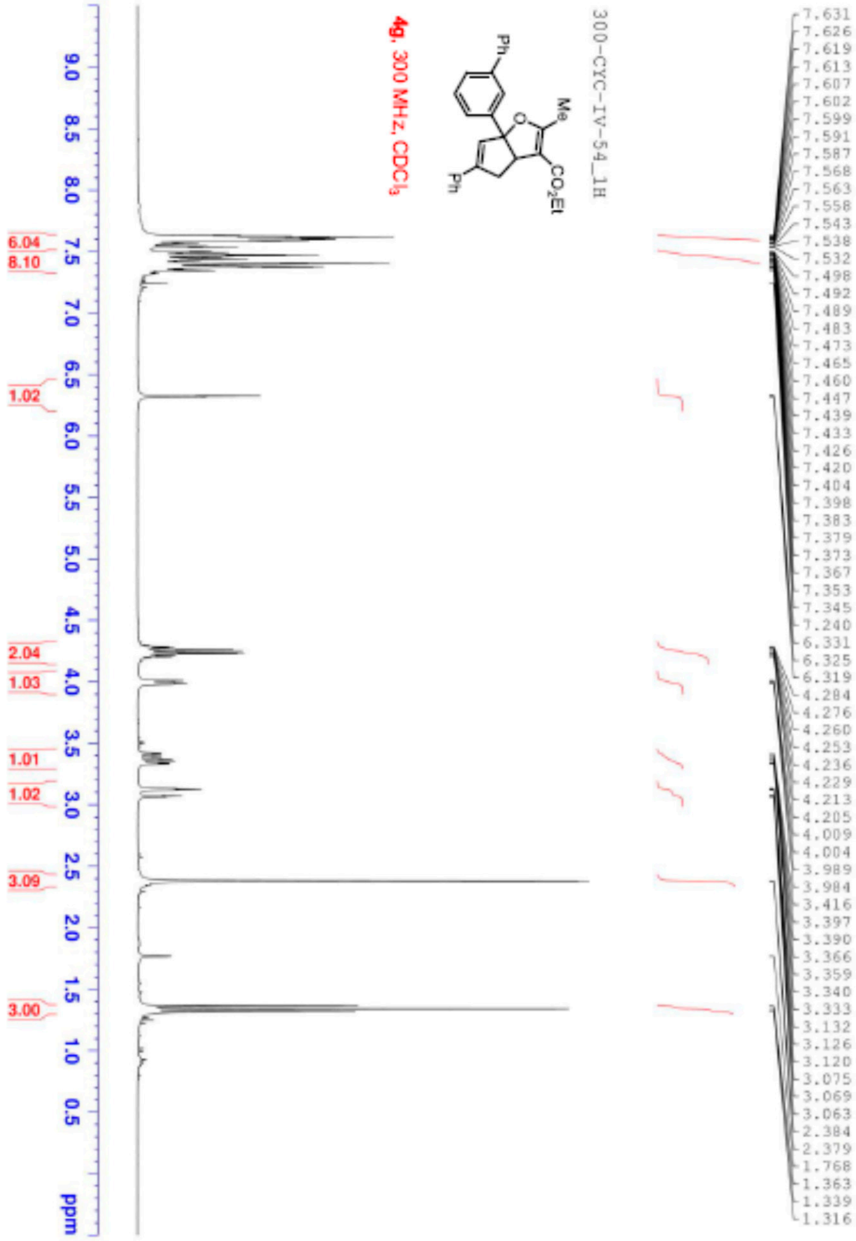

Figure S30. ^{1}H-NMR of **4g**.

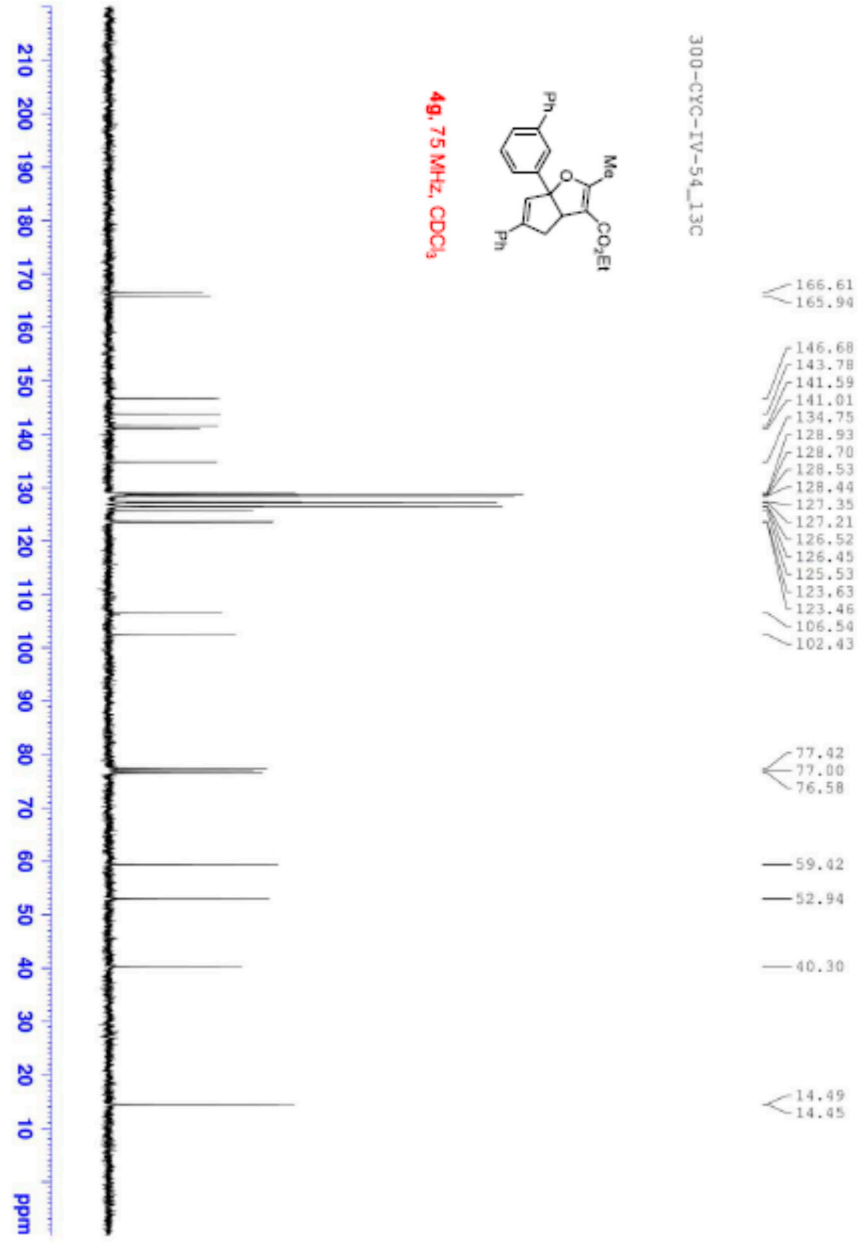

Figure S31. ^{13}C-NMR of **4g**.

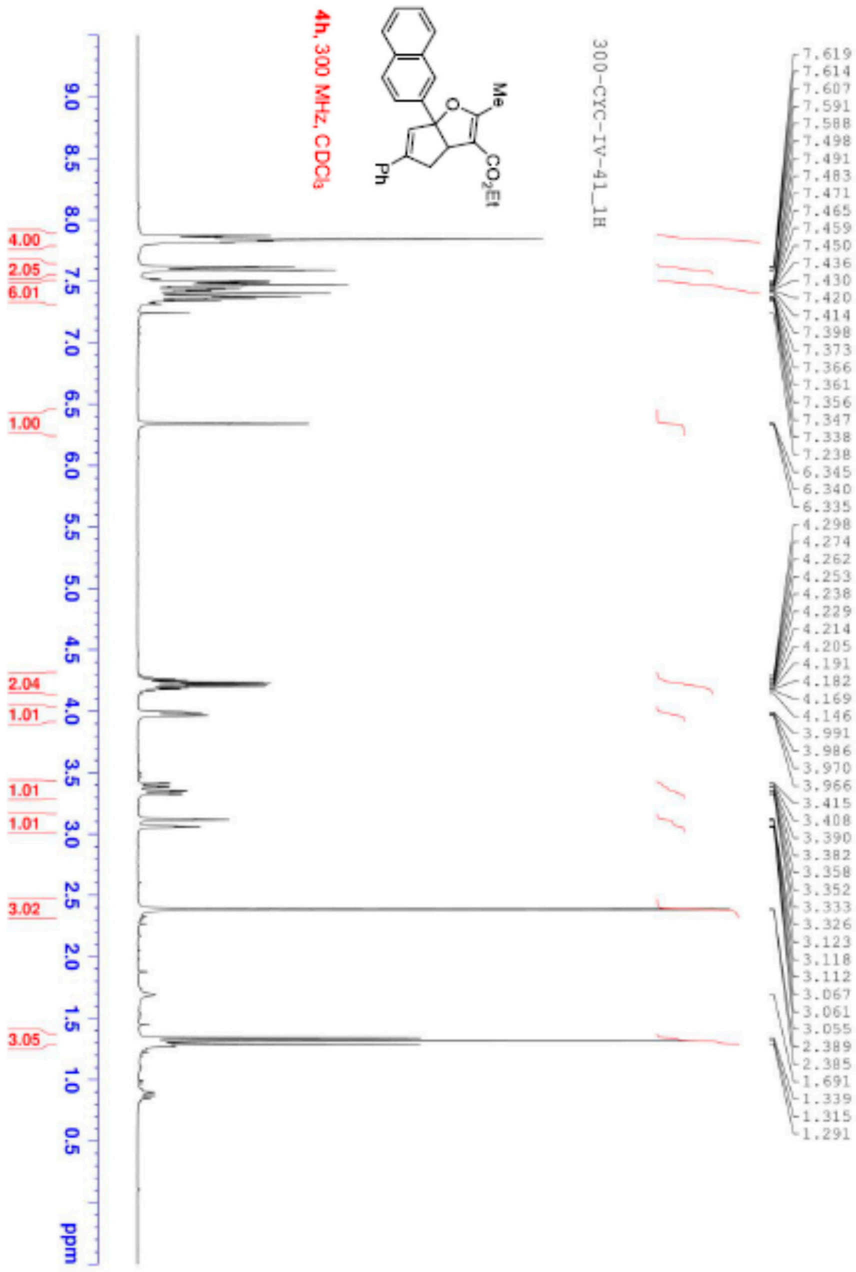

Figure S32. ^{1}H-NMR of **4h**.

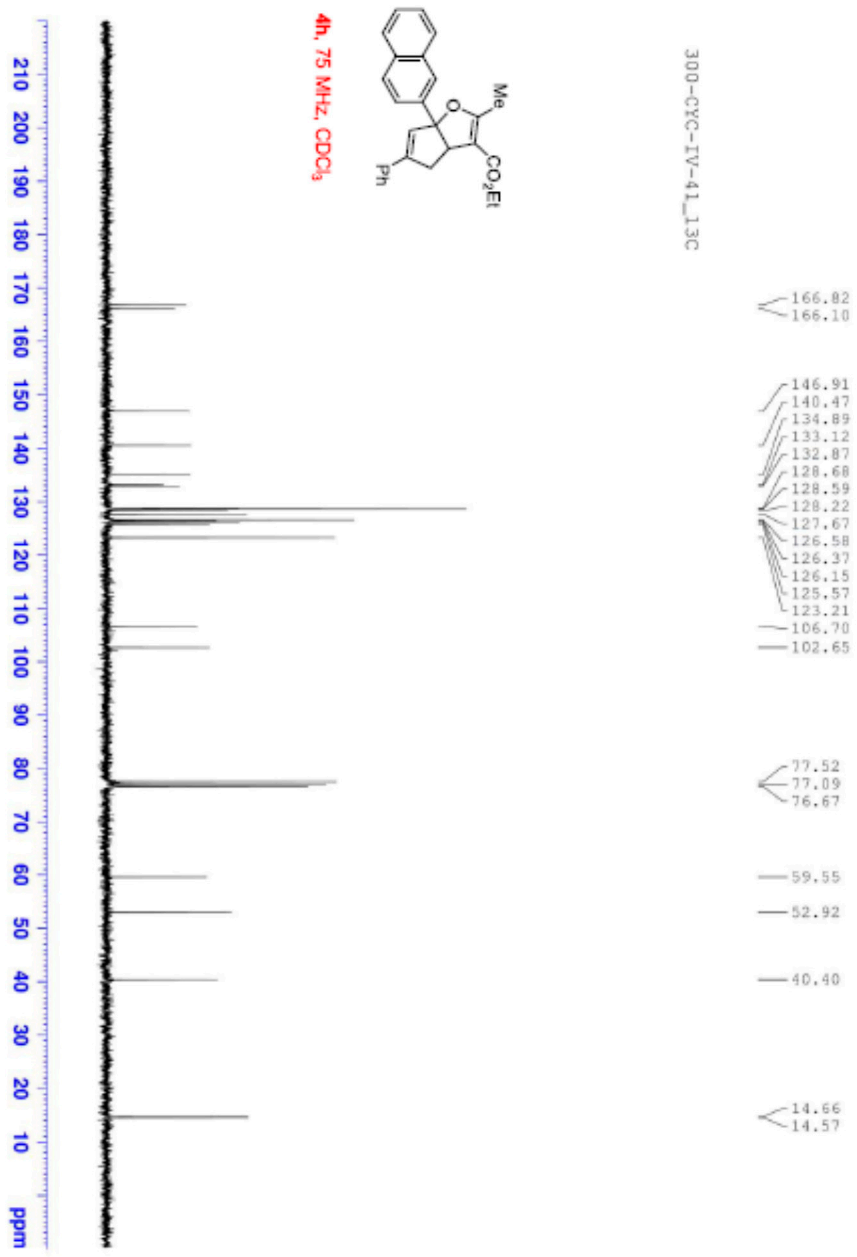

Figure S33. ^{13}C-NMR of **4h**.
